# The Solvothermal Method: An Efficient Tool for the Preparation of Ni-Based Catalysts with High Activity in CO_2_ Methanation

**DOI:** 10.3390/nano15171379

**Published:** 2025-09-06

**Authors:** Arkadii Bikbashev, Tomáš Stryšovský, Martina Kajabová, Zuzana Kovářová, Arati Prakash Tibe, Karolína Simkovičová, Robert Prucek, Aleš Panáček, Josef Kašlík, Patrizia Frontera, Kouřil Roman, Arian Grainca, Carlo Pirola, Libor Brabec, Zdeněk Bastl, Štefan Vajda, Libor Kvítek

**Affiliations:** 1 Department of Physical Chemistry, Faculty of Science, Palacký University Olomouc, 17. Listopadu 12, 77900 Olomouc, Czech Republic; arkadii.bikbashev01@upol.cz (A.B.); tomas.strysovsky@upol.cz (T.S.); martina.kajabova@upol.cz (M.K.); zuzana.kovarova@upol.cz (Z.K.); aratiprakash.tibe01@upol.cz (A.P.T.); karolina.simkovicova@jh-inst.cas.cz (K.S.); robert.prucek@upol.cz (R.P.); ales.panacek@upol.cz (A.P.); 2 Department of Nanocatalysis, J. Heyrovský Institute of Physical Chemistry, Czech Academy of Sciences, Dolejskova 2155/3, 18223 Prague, Czech Republic; stefan.vajda@jh-inst.cas.cz; 3 Czech Advanced Technology & Research Institute CATRIN, Regional Centrum of Advanced Technologies & Materials, Palacký University Olomouc, Slechtitelu 27, 78371 Olomouc, Czech Republic; josef.kaslik@upol.cz; 4 Department of Civil, Energy, Environmental and Material Engineering, Mediterranea University of Reggio Calabria, 89124 Reggio Calabria, Italy; patrizia.frontera@unirc.it; 5 Department of Biophysics, Faculty of Science, Palacký University Olomouc, Slechtitelu 27, 78371 Olomouc, Czech Republic; roman.kouril@upol.cz; 6 Department of Chemistry, University of Milan, Via Golgi 19, 20133 Milano, Italy; arian.grainca@unimi.it (A.G.); carlo.pirola@unimi.it (C.P.); 7 Center for Innovations in the Field of Nanomaterials and Nanotechnologies, J. Heyrovský Institute of Physical Chemistry, Czech Academy of Sciences, Dolejskova 2155/3, 18223 Prague, Czech Republic; 8 Department of Low-Dimensional Systems, J. Heyrovský Institute of Physical Chemistry, Czech Academy of Sciences, Dolejskova 2155/3, 18223 Prague, Czech Republic; zdenek.bastl@jh-inst.cas.cz

**Keywords:** heterogeneous catalysis, carbon dioxide, methanation, nickel oxide, nanoparticles, microparticles

## Abstract

Nickel and nickel oxide are widely used as heterogeneous catalysts in various processes involving the hydrogenation or reduction of organic compounds, and also as excellent methanation catalysts in the hydrogenation of CO_2_. As heterogeneous catalysis is a surface-dependent process, nickel compounds in the form of microparticles (MPs), and particularly nanoparticles (NPs), improve the catalytic activity of Ni-based catalysts due to their high specific surface area. Solvothermal synthesis, which has so far been neglected for the synthesis of Ni-based methanation catalysts, was used in this study to synthesize nickel and nickel oxide MPs and NPs with a narrow size distribution. Solvothermal synthesis allows for the control of both the chemical composition of the resulting Ni catalysts and their physical structure by simply changing the reaction conditions (solvent, temperature, or concentration of reactants). Only non-toxic substances were used for synthesis in this study, meaning that the whole synthesis process can be described as environmentally friendly. Solvothermally prepared Ni compounds were subsequently transformed into nickel oxide by means of high-temperature decomposition, and all of the prepared Ni-based compounds were tested as catalysts for CO_2_ methanation. The best catalysts prepared in this study exhibited a CO_2_ conversion rate of nearly 95% and a selectivity for methane close to 100%, which represent thermodynamic limits for this reaction at the used temperature. These results are commonly achieved with much more complex catalytic composites containing precious metals, while here we worked with pure nickel and its oxides, in the form of micro- or nanoparticles, only.

## 1. Introduction

Nickel and its compounds are among the most popular substances in heterogeneous catalysis [1,2]. As reflected in the thousands of studies and patents devoted to developing and studying various forms of nickel catalysts [3], up to 10% of the nickel produced in the world is used in the manufacturing of catalysts. The main field of utilization of nickel and nickel-based compounds in catalysis relates to various hydrogenation reactions [4,5].

One of the most popular and oldest catalysts based on pure nickel is Raney nickel, obtained in 1925 by melting nickel and aluminum together at 1200 °C [6,7]. This catalyst is mainly used for the hydrogenation and reduction of unsaturated organic compounds with hydrogen, but also to accelerate oxidation processes.

Nickel oxide (NiO) has high catalytic activity and is used as a catalyst in reactions related to the conversion of electrical energy to chemical energy (batteries) [8], the hydrogenation of compounds in the food industry [9], the polymerization of phenolic compounds [10], and the decomposition of toxic ozone in the environment [11]. It is also tested for the production of synthetic gas (methane) [12], the synthesis of carbon nanotubes, and natural gas purification processes.

Nickel hydroxide Ni(OH)_2_ is an unstable green powder that decomposes in a temperature range of 230–360 °C to NiO [13], meaning that it is not a usable compound for catalysis in high-temperature reactions. However, the decomposition product of this compound is NiO, which as stated previously is a very promising catalyst. Nickel metahydroxide NiO(OH) [14] is a black powder that decomposes into nickel oxide when heated to a temperature of about 600 °C. However, many non-stochiometric metahydroxides have been discovered. For instance, the non-stoichiometric nickel metahydroxide Ni(OH)_2_(NiOOH)_0.167_)_0.857_ prepared in our study was a yellow substance that decomposed to nickel oxide at a temperature of only 350–360 °C.

There are a wide variety of methods available for the synthesis of MPs and NPs of nickel compounds: the thermal decomposition of precursors prepared by means of the chemical precipitation method [15,16], sonochemical synthesis [17,18], chemical vapor deposition [19,20], biosynthesis [21,22], the sol–gel method [23,24], etc. Unfortunately, many published preparation methods are unsuitable for industrial production due to a lack of scalability.

All nickel compounds used in the current study were obtained using solvothermal synthesis based on the hydrolysis (and reduction) of soluble nickel compounds [25,26,27,28,29,30,31]. Solvothermal reactions are interesting in their similarity to actual production processes in industry, since they are carried out in autoclaves (special containers that can withstand high pressure). Moreover, these reactions are relatively simple to perform and provide good and reproducible results in producing highly dispersed micro- and nanoparticles of inorganic substances. Some disadvantages of solvothermal synthesis include its duration and the need for high temperature and pressure during the reaction, which can easily be addressed by using an autoclave reactor. Meanwhile, there is minimal to no need to use toxic or environmentally hazardous solvents and other chemicals in the process, meaning that solvothermal methods can, in many cases, be labeled as environmentally friendly.

The modified Fischer–Tropsch [32,33] reaction involves the conversion of carbon dioxide via hydrogenation to form hydrocarbons and their derivatives. In the case of the preferential formation of CH_4_, the process is called methanation and proceeds according to the classical Sabatier scheme [34,35,36]:

CO_2_ + 4H_2_ → CH_4_ + 2H_2_O

Due to the high thermodynamic stability of CO_2_, the methanation process is complicated to implement in practice. A high temperature and effective catalysts are needed for its application on an industrial scale to remove excess carbon dioxide from the atmosphere and obtain valuable energy-rich compounds, e.g., methane.

Nickel compounds are promising catalysts for the methanation reaction, with the obtained conversion and methane selectivity reaching thermodynamic limits [37,38,39]. The main goal of this study, and the basis of its novelty, is the verification of the usability of the solvothermal reaction for producing effective Ni-based methanation catalysts, as the solvothermal method is easily scalable from laboratory experiments up to industrial production. Additionally, using only nickel salt, sodium acetate, ethylene glycol, and ethanol as solvents allows this method to be labeled as environmentally friendly in comparison with traditionally used precipitation methods. Based on the selection of the source compound of nickel and the solvent for the reaction itself, a series of compounds, including metallic Ni, Ni hydroxide, Ni metahydroxide, and Ni oxide, were prepared and subsequently tested as catalysts for CO_2_ methanation. The study’s second goal was to explore the influence of the thermal treatment of the primary prepared Ni-based compounds on their catalytic efficiency.

## 2. Experimental

### 2.1. Materials

Ni(Cl)_2_·6H_2_O (98.5%), ethylene glycol (EG, 99.98%), ethanol (Et, 95%), and CH_3_COONa·3H_2_O (NaAc·3H_2_O, 99.8%) were obtained from Lach-Ner, Neratovice, Czech Republic. Silica gel (SG) was obtained from Penta Chemicals Unlimited, Prague, Czech Republic, while polyethylene glycol 1500 (PEG 1500) was purchased from Fluka Chemika (Buchs, Switzerland). Deionized water (DW, 18 MΩ·cm, Millipore, Burlington, MA, USA) was used to prepare all aqueous solutions.

### 2.2. Catalyst Preparation

The precatalysts (Ni-based compounds ranging from pure Ni to Ni metahydroxides) were prepared using a solvothermal method similar to that described in previous publications [40,41,42]. The scheme of preparation used was as follows: NiCl_2_·6H_2_O (2.38 g, 0.01 mol) was dissolved in 80 mL of EG (or 40 mL EG + 40 mL Et) in a 150 mL beaker at room temperature, followed by the addition of CH_3_COONa·3H_2_O (5.96 or 11.92 g). After 10 min of stirring (700 rpm), PEG 1500 (3 g) was added, and the mixture was stirred for 30 min. Finally, the mixture was placed in a 200 mL Teflon autoclave and left to react at 200 °C for 8 h. Products of this primary preparation step were separated via centrifugation and dried (vacuum, 0.1 bar, 60 °C/8 h). After this step, the final oxides for catalytic testing were obtained by means of the thermal decomposition of the prepared precursors at elevated temperature in the laboratory furnace under an air atmosphere. The specific conditions for preparing all tested samples are presented in Table 1.

### 2.3. Instruments and Methods of Characterization

Thermogravimetric analysis was conducted with TG/DSC SDT 650 (TA Instruments, New Castle, DE, USA). The crystal structure and chemical composition of the catalysts were studied by means of powder X-ray diffraction using an X’pert Pro (Malvern PANalytical, Malvern, UK) device. XRD analyses were conducted using a CoKa-radiation source in the 5–105° 2 Theta range and a total measurement time of 128 min/sample. The reduction process of the NiO samples was monitored using the same X’pert Pro device with a mounted XRK 900 reaction chamber (Anton Paar, GmbH, Graz, Austria): approximately 80 mg of the sample was placed in the sample holder, heated to 50 °C, and diffraction was measured for 10 min. Subsequently, the average diffraction over 10 min was measured at 25 °C intervals as temperature increased until the material was completely reduced to nickel metal. A measurement time of 10 min per scan was selected to allow for the detection of relatively rapid structural and phase changes. Additionally, the angular measurement range was narrowed to 5 to 80° 2 Theta to improve the signal-to-noise ratio. The reduction was carried out in a hydrogen atmosphere (purity 99.9999% H_2_) at atmospheric pressure with a flow rate of 45 mL/min.

Surface area, temperature-programmed H_2_ reduction (TPR-H_2_), and temperature-programmed CO_2_ desorption (TPD-CO_2_) characterizations were performed on a 3-flex Micromeritics device from Micromeritics (Norcross, GA, USA). All analyses were carried out in a temperature range of 25–600 °C with a heating rate of 10 °C/min. Determination of PSD was conducted using the BJH-A model with corrections via the Kruk–Jaroniec–Sayari method with data obtained from the adsorption part of the measured isotherms. 

The SEM images were obtained using a Scios 2 Dual Beam microscope at an accelerating voltage of 5 kV and Phenom Pro X (both from Thermo Fisher SCIENTIFIC, Waltham, MA, USA), while the TEM images were obtained using a Tecnai G2 F20 microscope (FEI Technologies, Hillsboro, OR, USA) with an Eagle 4K CCD camera (FEI Technologies, Hillsboro, OR, USA) and TEM JEOL 2100, 200 kV (JEOL, Peabody, MA, USA). Surface elemental mapping of the spent catalysts was conducted using the EDS method in combination with high-resolution transmission electron microscopy (HRTEM) on an FEI TITAN 60–300 kV microscope (Thermo Fisher Scientific Inc., Waltham, MA, USA).

The sample’s X-ray photoelectron spectra (XPS) were measured using a modified ESCA 3 MkII multi-technique spectrometer equipped with a hemispherical electron analyzer operated in fixed transmission mode. Al Kα radiation was used for electron excitation. The binding energy scale was calibrated using the Au 4f_7/2_ (84.0 eV) and Cu 2p_3/2_ (932.6 eV) photoemission lines, and the pressure in the XPS analysis chamber during spectrum acquisition was 6 × 10^−9^ mbar. The samples were spread on an aluminum surface, and the spectra were collected at a takeoff angle of 45° in relation to the macroscopic surface’s normal. High-resolution spectra of Ni 2p, Si 2p, O 1s, and C 1s photoelectrons were measured, and the spectra of Ni 2p_3/2_ photoelectrons were curve fitted after subtraction of the Shirley background [43] using the Gaussian−Lorentzian line shape and nonlinear least-squares algorithms (CasaXPS ver. 2.3.15 software [44]). Binding energies were obtained with reference to the C 1s peak of adventitious carbon at 285.0 eV, providing 103.4 eV for the Si 2p peak. The elemental concentrations were quantified by correcting the photoelectron peak intensities for their cross sections [45] and the analyzer transmission function.

Raman spectra were recorded using a Raman microscope DRX (Thermo Fisher Scientific Inc., Waltham, MA, USA) with an excitation laser wavelength of 780 nm and an estimated spot size of 3.1 μm. For the experiments, a 400 line/mm grating with a resolution of 5.0 cm^−1^ FWHM was used, and the spectral dispersion was 2 cm^−1^ per CCD pixel element. Laser power was chosen for each sample individually according to its photosensitivity (4.5–10.0 mW). Each spectrum was averaged from 250 scans, with the exposure time for one spectrum being 2 s.

The catalytic reactions were studied using the flow reactor Micro EFFI PID from PID Eng&Tech (Madrid, Spain), with the reaction tube being 4 mm in diameter. The analysis of reaction products was performed using gas chromatography with an Agilent 7890B equipped with a TCD detector and a mass spectrometer, the Agilent 5977B (Santa Clara, CA, USA). The gas reaction mixture and other conditions of the conducted reactions are stated in the next section for the individual studied cases.

### 2.4. Catalytic Performance

#### 2.4.1. Evaluation of Catalysis Efficiency

The conversion of CO_2_ ($X_{CO2}$, Equation (1)), selectivity for CO ($S_{CO}$, Equation (2)), selectivity for CH_4_ ($S_{CH4}$, Equation (3)), reaction yield of CH_4_ ($\eta_{CH4}$, Equation (4)), and space–time yield (STY_CH4_, Equation (5)) were calculated for each catalyst. STY_CH4_ indicates the amount of methane produced per unit weight of the catalyst.

(1) $$X_{CO2}=\left(\frac{\left[CO\right]+\left[{CH}_{4}\right]}{\left[{CO}_{2}\right]+\left[CO\right]+\left[{CH}_{4}\right]}\right)\times 100\%$$

(2) $$S_{CO}=\frac{\left[CO\right]}{\left[CO\right]+\left[{CH}_{4}\right]}\times 100\%$$

(3) $$S_{CH4}=\frac{\left[{CH}_{4}\right]}{\left[{CH}_{4}\right]+\left[CO\right]}\times 100\%$$

(4) $$\eta_{CH4}=\frac{\left[{CH}_{4}\right]}{\left[{CH}_{4}\right]+\left[{CO}_{2}\right]+\left[CO\right]}\times 100\%$$

(5) $${STY}_{{CH}_{4}}=\frac{F_{{CO}_{2}}\times_{{CO}_{2}}s_{{CH}_{4}}}{m_{cat}}$$

F_CO2_ is the molar flow rate of CO_2_ [mmol·h^−1^] and m_cat_ is the weight of the catalyst [g].

#### 2.4.2. Catalytic Tests—Temperature Ramp

The catalytic mixture for this test experiment was obtained by mixing 100 mg of NiO and 150 mg of silica. The catalytic test was conducted with activation at 300 °C for 2 h at 1 bar of H_2_ atmosphere, and the temperature ramp used started at 350 °C and finished at 475 °C with 25 °C intervals. The total flow of reaction gases was 66 mL/min at a ratio of H_2_ to CO_2_ of 4:1 (CO_2_, 6 mL/min, H_2_, 24 mL/min, He, 36 mL/min, equal to 55% of the gaseous mixture). The reaction pressure was adjusted to 30 bar. The reaction time for each temperature step was 3 h after reaching the desired temperature.

#### 2.4.3. Catalysis Conditions for Stability Tests

The main study of the catalytic performance and stability of the prepared catalysts was conducted under the following conditions:

(1) The amount of catalyst samples used was 100 mg NiO/Ni/Ni(OH)_x_ + 150 mg SG;
(2) Activation of the catalyst was performed at 300 °C, 1 bar, for 2 h using a pure H_2_ atmosphere;
(3) Catalysis was conducted at 450 °C, 30 bar, for 21 h, but two samples (Ni(a) and NiO600(b)) were also tested for 42 h; CO_2_ flow was adjusted to 6 mL/min, H_2_ flow was adjusted to 24 mL/min (ratio of H_2_ and CO_2_ 4:1), that of He was adjusted 36 mL/min (55% of the gaseous mixture).

However, in all presented graphs from stability catalytic experiments, the activation time is not included in the overall catalytic time, as the initial moment of catalysis (t = 0 h) was taken as the moment at the end of activation when reaching a temperature of 450 °C.

## 3. Results and Discussion

### 3.1. TG/DSC Study of Ni-Based Precursors

**Figure 1:**
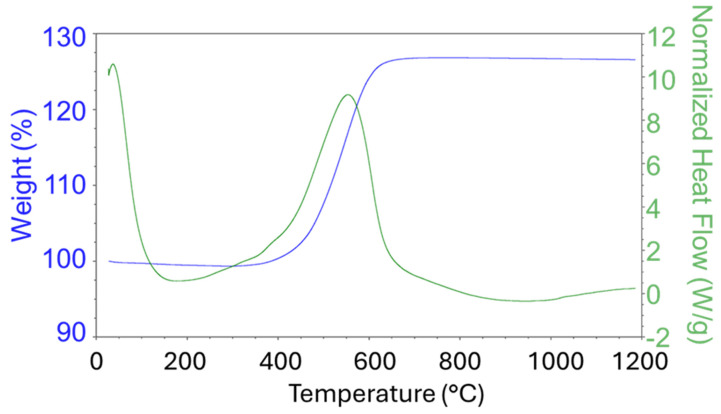
TG/DSC graphs of Ni(a) measured under an oxidation atmosphere (air).

A sample of the precursor Ni(a) was tested in the temperature range 40–1200 °C (Figure 1). As can be seen from the DSC graph, the phase transformation occurred once, at about 550 °C. The TG graph demonstrates that the sample grew in weight by about 27% compared to its initial mass at this temperature. XRD confirmed that a pure Ni precursor was initially prepared by means of the solvothermal method, and it was transformed into NiO due to oxidation at elevated temperatures. The obtained data corresponds to the stoichiometric equation of the thermal oxidation of nickel:

Ni+1/2O_2_ = NiO (M(NiO)/M(Ni) = 74.7 × 100%/58.7 = 27.25%)

Based on this measurement, two nickel oxide precatalysts were prepared from the Ni(a) precursor at 600 and 1000 °C (NiO600(a) and NiO1000(a)).

**Figure 2:**
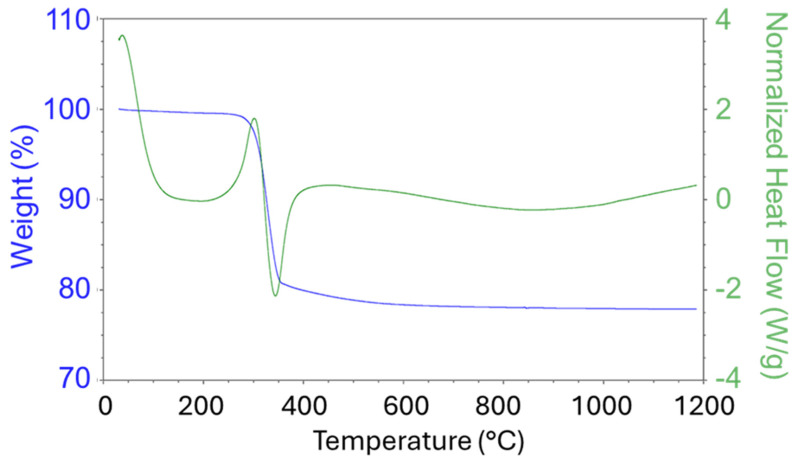
TG/DSC graphs of Ni(OH)_2_(b) measured under an oxidation atmosphere (air).

A sample of the Ni(OH)_2_(b) precursor was studied at the temperature range 40–1200 °C (Figure 2). As can be seen from the DSC graph, the phase transformation occurred at about 340 °C. The TG graph demonstrates that the sample lost about 20% of its initial mass. XRD confirmed that α-Ni(OH)_2_ was initially prepared by means of the solvothermal method, which was transformed into NiO at elevated temperatures. The analysis data corresponds to the stoichiometric equation of the thermal decomposition of nickel hydroxide:

Ni(OH)_2_ = NiO+H_2_O (M(H_2_O)/M(Ni(OH)_2_) = 18 × 100%/92.7 = 19.41%)

Based on this diagram, two nickel oxide precatalysts were prepared from this precursor by means of thermal decomposition at 400 and 600 °C (NiO400(b) and NiO600(b)).

**Figure 3:**
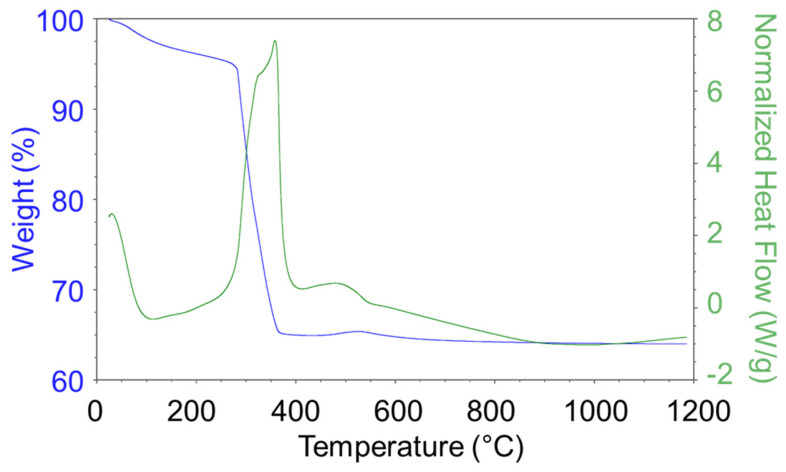
TG/DSC graphs of the NiO(OH)_x_(c) decomposition process (air atmosphere).

The NiO(OH)_x_(c) precursor was studied under the same conditions as the two previous precursors at a temperature range of 40–1200 °C (Figure 3). As can be seen from the DSC graph, the phase transformation occurred at about 350 °C, which was a slightly higher temperature in comparison with the Ni(OH)_2_(b) precursor. The TG graph shows that the sample lost about 35% of its initial mass at this temperature. XRD indicated that (Ni(OH)_2_(NiOOH)_0.167_)_0.857_ was initially prepared and then thermally transformed into NiO. Based on this diagram, two nickel oxide precatalysts were prepared from this precursor by means of thermal decomposition at 400 and 600 °C (NiO400(c) and NiO600(c)).

### 3.2. XRD Analysis of Precursors and Precatalysts

XRD analysis of precursors showed that Ni(a) mostly consisted of a cubic structure of Ni with a trace content of a hexagonal structure. In turn, the Ni(OH)_2_(b) precursor consisted of 100% *α*-Ni(OH)_2_, while the NiO(OH)_x_(c) precursor displayed a non-stoichiometrically complex hydroxide structure (Ni(OH)_2_(NiOOH)_0.167_)_0.857_. Therefore, the amount of sodium acetate used during synthesis was insufficient to completely convert NiCl_2_ into stoichiometric nickel hydroxide Ni(OH)_2_.

All nickel oxide precatalysts (NiO600(a), NiO1000(a), NiO600(b), NiO600(c)) prepared at temperatures of 600 and 1000 °C contained 100% NiO. However, a temperature of 400 °C was not high enough for the complete conversion of hydroxide structures (Ni(OH)_2_(b) and NiO(OH)_x_(c)) into nickel oxides, and the samples NiO400(b) and NiO400(c) contained 0.8% Ni. The full results of the XRD measurements are presented in Table 2 and Figure S1a–h.

In addition, XRD analysis of the reduction of the prepared NiO600(b) and NiO600(c) oxides under elevated temperature was carried out. This experiment showed that the complete reduction of the oxide to Ni metal occurred as early as 300 °C (see Figure S2a,b), and therefore this temperature was chosen for the activation of the precatalyst in the CO_2_ methanation reactor.

### 3.3. Temperature-Programmed H_2_ Reduction (TPR-H_2_) and Temperature-Programmed Desorption of CO_2_ (TPD-CO_2_) Characterization of Precatalysts

The TPR-H_2_ and TPD-CO_2_ diagrams of the selected precatalyst samples show their interaction with both test gases at an increased temperature (temperature range: 20–600 °C).

**Figure 4:**
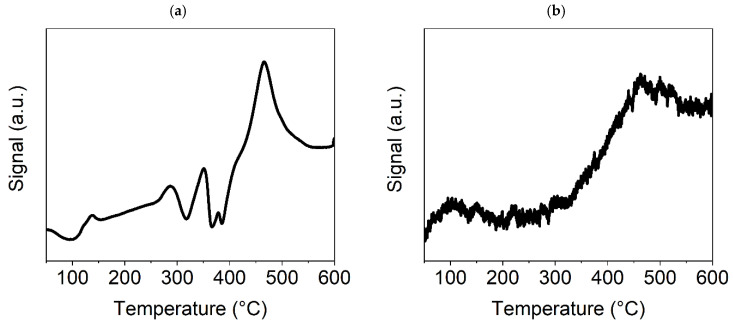
TPR-H_2_ (**a**) and TPD-CO_2_ (**b**) graphs for the Ni(a) sample.

In Figure 4a, for the TPR-H_2_ of the Ni(a) sample, three small peaks can be seen in the temperature range of 250–400 °C, indicating a consecutive reduction of the Ni surface oxides, formed during manipulation with this catalyst before the catalytic experiment in the air. The fourth more pronounced peak at about 450 °C is likely connected with the chemisorption of H_2_ on the surface of metallic Ni particles. Based on this experiment, an activation temperature of 300 °C was chosen for the conducted catalytic experiments. The TPD-CO_2_ method showed strong sorption of CO_2_, as demonstrated by a high and broad peak starting at about 450 °C (Figure 4b). In addition to this main peak, the graph also shows a significantly smaller peak at about 100 °C, which corresponds to weak basic sites on the catalyst surface, in contrast to the main peak at about 460 °C, which is associated with CO_2_ adsorption on medium basic sites. Since the signal originating from CO_2_ desorption does not decrease with increasing temperature from the main peak value reached, it can be concluded that the basicity of these sites is very close to that of the strong basic sites on which CO_2_ adsorption is the highest [46]. As the medium basic sites are the most important for the efficient catalytic transformation of CO_2_, a temperature of 450 °C was identified as being optimal for catalyst stability and performance tests. This temperature was also confirmed by catalytic preliminary tests (see Section 3.7.1).

**Figure 5:**
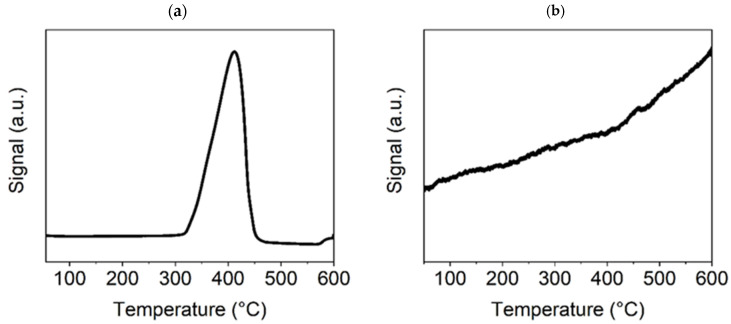
TPR-H_2_ (**a**) and TPD-CO_2_ (**b**) graphs for the Ni600(b) sample.

In Figure 5a, for the TPR-H_2_ of the NiO600(b) sample, there is only one high peak at approximately 400 °C, which indicates the reduction of this sample to metallic nickel; additional XRD tests (see Section 3.2) confirmed the complete reduction of Ni oxides to metallic Ni at a temperature of 300 °C. The presence of metallic nickel in the sample usually improves its catalytic properties. However, this will be discussed further in Section 3.7.2, which is focused on the catalytic activity of the Ni-based catalysts. The TPD-CO_2_ graph (Figure 5b) demonstrates no peak, only a linearly increasing line indicating the existence of nonspecific basic sites (discrete distribution of sites according to their basicity) on the surface of Ni oxide. However, the growing signal from CO_2_ desorption with increasing test temperature indicates that the adsorption of CO_2_ on the NiO surface could be sufficient for catalysis to proceed at temperatures above 400 °C, when the curve breaks and becomes steeper.

**Figure 6:**
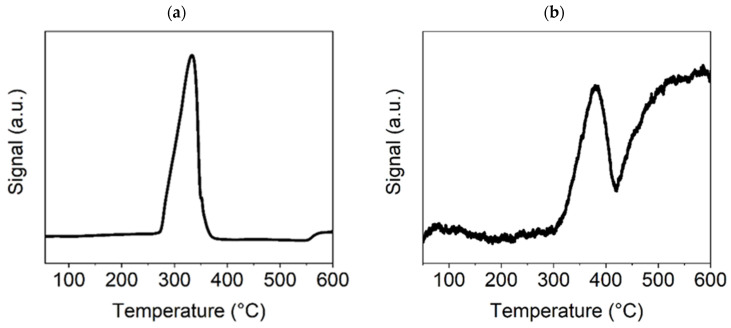
TPR-H_2_ (**a**) and TPD-CO_2_ (**b**) graphs for the Ni(OH)_2_(b) sample.

In Figure 6a, for the TPR-H_2_ experiment with Ni(OH)_2_(b), the presence of only one strong peak indicates the reduction of hydroxide to metallic nickel at a temperature of approximately 330–340 °C, which is lower than was observed in the case of the NiO600(b). This may have been caused by the higher specific surface area for this sample due to its smaller particles, leading to a rapid reduction to metallic Ni. As was shown previously, from the results of the thermogravimetric test (Figure 2), at approximately this temperature, nickel hydroxide decomposes to oxide and subsequently is reduced by the H_2_ to metallic Ni. The CO_2_-TPD diagram (Figure 6b) shows three CO_2_ desorption peaks. The peak slightly below 100 °C corresponds to weak basic sites on the catalyst surface. The sharp peak at around 380 °C corresponds to a high concentration of medium basic sites on the surface, which is very important for the Ni-based methanation catalyst’s catalytic efficiency. The following broad peak at around 500 °C corresponds to strong basic sites, the concentration of which is also likely to be high because, after reaching the maximum value, desorption does not decrease with a further temperature increase. The results for both temperature-programmed experiments indicate that this Ni compound is suitable for the catalysis of the methanation process.

TPR and TPD experiments with the selected precatalysts showed essential differences in their reducibility and sorption properties in relation to CO_2_. Ni(a) is a pure Ni metal compound, meaning that in a H_2_ atmosphere, it is reduced only in small areas on its surface, which were formed during the preparation steps conducted in an air atmosphere. The other two precatalysts are reduced in the H_2_ atmosphere to metallic Ni, but hydroxide is reduced more easily than oxide, as can be seen from the TPR-H_2_ graphs of the Ni(OH)_2_(b) and NiO600(b) samples. Additionally, hydroxide can more effectively (and reversibly) sorb CO_2_ in comparison with oxide and metallic Ni, which is an essential property in the hydrogenation of CO_2_ from the point of view of catalytic activity. Comparison of these CO_2_-TPD graphs shows that only in the case of the Ni(OH)_2_(b), the measurable amount of CO_2_ is adsorbed on the medium basic sites, which are commonly connected with the O defects on the surface of the catalyst, which are important for high catalytic activity in CO_2_ hydrogenation [47]. Therefore, the TPR and TPD experiments provided useful information for preparing an effective Ni-based catalyst for CO_2_ methanation, revealing Ni hydroxide to be the best Ni catalyst, with this assumption being confirmed by subsequent catalytic tests.

### 3.4. Electron Microscopy of the Ni-Based Catalysts

#### 3.4.1. SEM/TEM Before Catalysis

**Figure 7:**
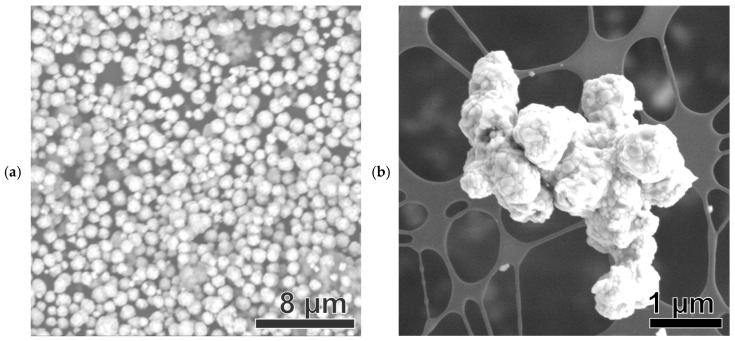

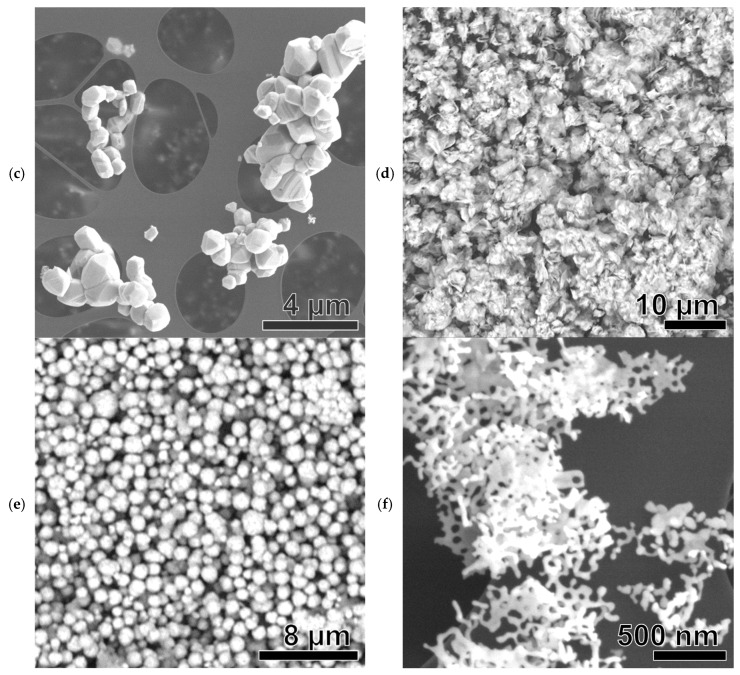

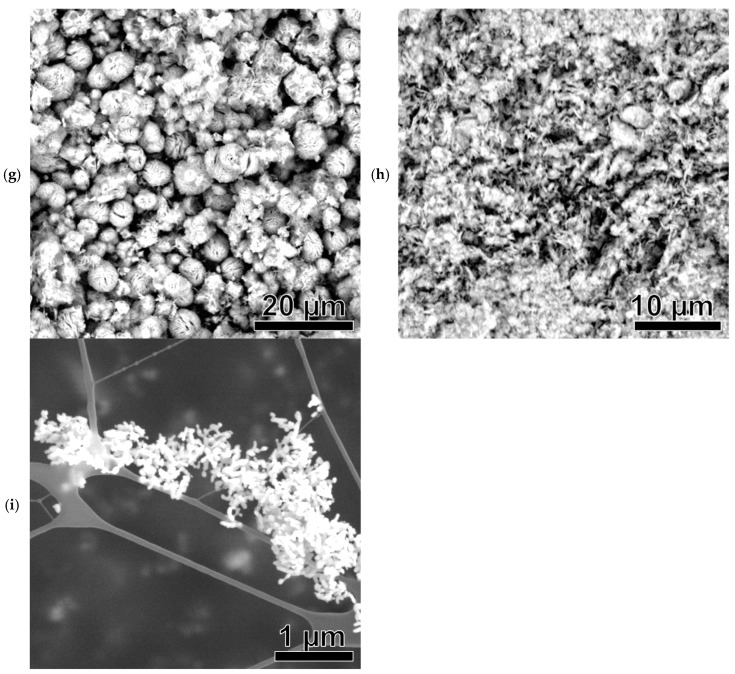
SEM images of Ni-based samples: Ni(a) (**a**), NiO600(a) (**b**), NiO1000(a) (**c**), Ni (OH)2(b) (**d**), NiO400(b) (**e**), NiO600(b) (**f**), NiO(OH)x(c) (**g**), NiO400(c) (**h**), and NiO600(c) (**i**).

The SEM image of the Ni(a) sample in Figure 7a shows the presence of spherical MPs 0.5–2 μm in size. Several particles of polyhedral form (octahedron, dodecahedron, etc.) are also present, as confirmed by the TEM image in Figure S3a.

The SEM image of the NiO600(a) sample (a derivative of Ni(a)) shows a structure differing from that of the original precursor Ni(a), comprising clusters with spherical-like MPs with a diameter of about 500–1000 nm (Figure 7b). These clusters, in turn, mostly consist of shapeless, and to some extent disk-like, NPs about 50–100 nm in size. The TEM image in Figure S3b confirms this structure.

The SEM image in Figure 7c depicts NiO1000(a), a derivative of Ni(a). In this case, clusters assembled from an irregular polyhedral can be seen, 0.1–1.5 μm in size. Sphere-like structures are not observable. The TEM image in Figure S3c confirms the SEM analysis data.

In the case of the SEM image of Ni(OH)_2_(b) in Figure 7d, it can be observed that massive clusters with a rough structure of peculiar microsheets about 2–3 μm in size are present. The TEM image in Figure S3d shows the multi-layered nature of these clusters.

NiO400(b) (a derivative of Ni(OH)_2_(b)), as shown in the SEM image in Figure 7e, has a structure differing from that of the original hydroxide, consisting of spherical-like MPs with a diameter of about 0.5–3 μm. In the corresponding TEM image (Figure S3e), plenty of spherical and cylindrical NPs around 50–100 nm in size can be seen, making up the spherical-like MPs.

NiO600(b) (a derivative of Ni(OH)_2_(b)) has a significantly different structure compared to the particles forming it (Figure 7f), with these particles creating a net of several microns in size with holes less than 100 nanometers in diameter. The TEM image in Figure S3f also shows the continuous nature of this structure.

NiO(OH)_x_(c) has rounded MPs with a broad size distribution ranging from 200 nm to about 3 μm (Figure 7g). The fibrous, tangled nature of these particles is evident. The TEM image in Figure S3g shows a round, needle-like structure resembling a hedgehog. The particles display some stabilization but are also prone to cluster formation.

NiO400(c) (a derivative of NiO(OH)_x_(c)) has the shape of a vast, endless cluster with a rough, needle-like surface (Figure 7h). However, in the TEM image in Figure S3h, it is possible to identify individual MPs with a size of about 5–8 μm and a hedgehog-like shape.

NiO600(c) (a derivative of NiO(OH)_x_(c)) has a very different structure from the previous sample. Structurally, the sample is a set of small cylindrical rods with a length of less than 100 nm (Figure 7i). This structure is confirmed in the TEM image in Figure S3i.

SEM EDX analysis (Figure S4a–c) confirmed the precatalysts’ elemental composition, containing only Ni and O. Carbon is also detectable on some spectra, which probably originates from residues of PEG 1500 used in the preparation method as a stabilizer.

In conclusion, the SEM images show that nickel oxides, after calcination, do not display the same structure as primary nickel or nickel hydroxide particles. Moreover, the morphology of MPs and NPs of oxides also changes with increased calcination temperature. These changes affect the catalytic activity of prepared nickel catalysts, as explored later in this paper.

#### 3.4.2. SEM After Catalysis

**Figure 8:**
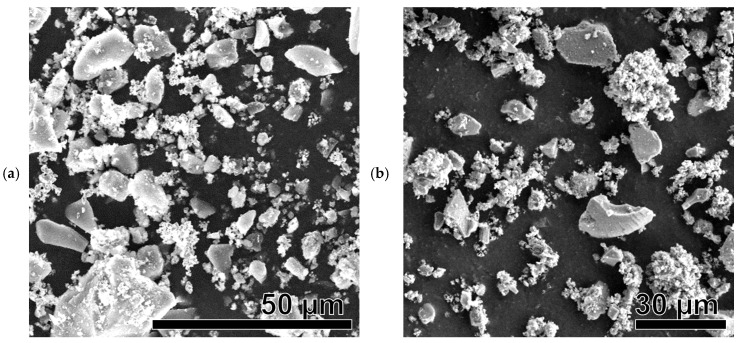

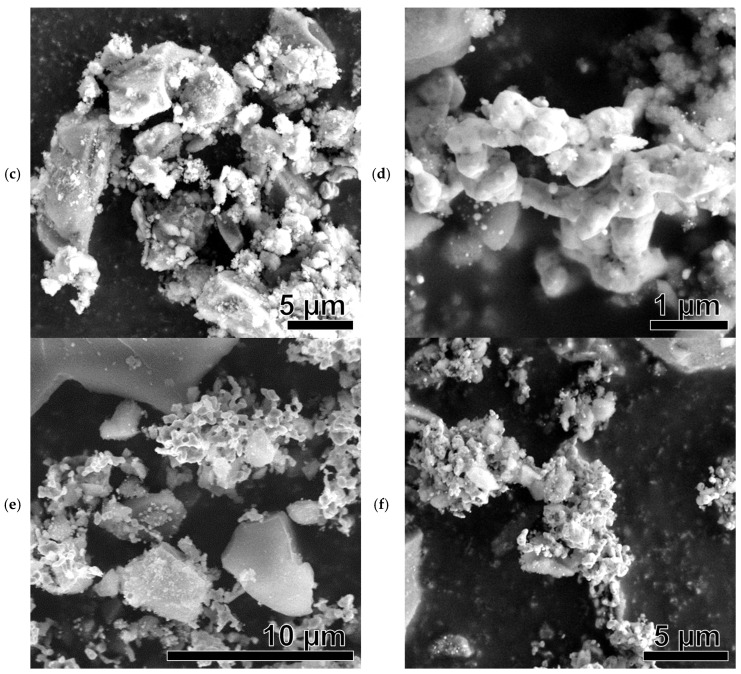

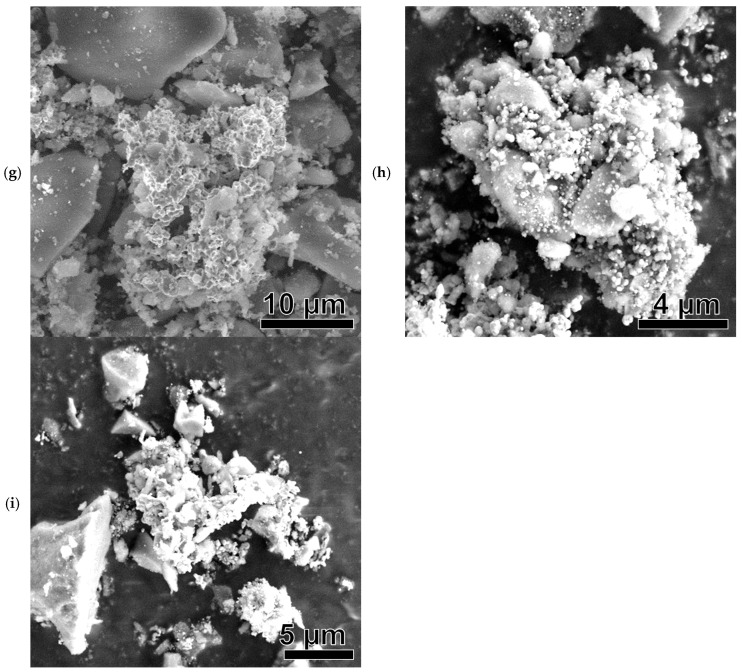
SEM images of Ni-based samples after catalysis: Ni(a)* (**a**), NiO600(a)* (**b**), NiO1000(a)* (**c**), Ni (OH)2(b)* (**d**), NiO400(b)* (**e**), NiO600(b)* (**f**), NiO(OH)x(c)* (**g**), NiO400(c)* (**h**), and NiO600(c)* (**i**).

The SEM images in Figure 8a–i reveal a cardinal change in the morphology of the Ni-based catalysts after the catalytic reaction. Amorphous MPs can be observed to be several microns in size. The observed structures are therefore different from those of the original particles before catalysis. It can be safely argued that this change is a result of thermal stress and chemical modifications of primary Ni-based particles during catalysis; particles lose their shape and aggregate to form more giant structures, which may be the reason for the observed gradual decrease in catalytic activity over the course of the experiment (see Section 3.7). However, in Figure 8f–i, traces of the original morphology of the particles can be observed (net-like, sphere-like, needle-like, and rod-like particles).

All SEM images show brighter, larger (several tens of micrometers) silica particles that did not participate in the catalytic reaction. They were used as unreactive supporting material to avoid the sintering of Ni-based catalytic particles.

### 3.5. Surface Area and Pore Volume Characterization

The values of the surface area (m^2^/g) and pore volume (cm^3^/g) are presented in Table 3, and graphs of the measured isotherms are provided in Figure S5a,b.

The data presented shows a clear trend, where the highest surface area values are primarily observed for the prepared Ni-based compounds. Pure metal Ni shows the lowest surface area value, at only 7.03 m^2^/g, while metahydroxide has a medium value of 70.07 m^2^/g and Ni(OH)_2_ has the highest value of 83.99 m^2^/g. Thermal treatment reduced these values, with higher temperatures having a greater impact. Therefore, the sample Ni1000(a), formed via the oxidation of Ni(a) at 1000 °C, shows a surface area value of only 1.09 m^2^/g. The observed values for specific surface area are consistent with the electron microscopic observations. The lowest values of this quantity are connected with the smooth spherical microparticles of pure Ni, which, after oxidation at elevated temperatures, form larger aggregates with a smaller surface area. The existence of the aggregates is apparently a reason for the highest values of pore size for these samples with the smallest specific surface area. On the other hand, samples with the highest surface area are formed by the imperfectly developed nanocrystalline particles of Ni(OH)_2_ or of the non-stochiometric Ni metahydroxide NiO(OH)_x_, and their pore size is undoubtedly caused by the irregular structure of particles, which improves the value of specific surface area and consequently the catalytic activity.

### 3.6. X-Ray Photoelectron Spectroscopy of Post-Catalytic Samples

**Figure 9:**
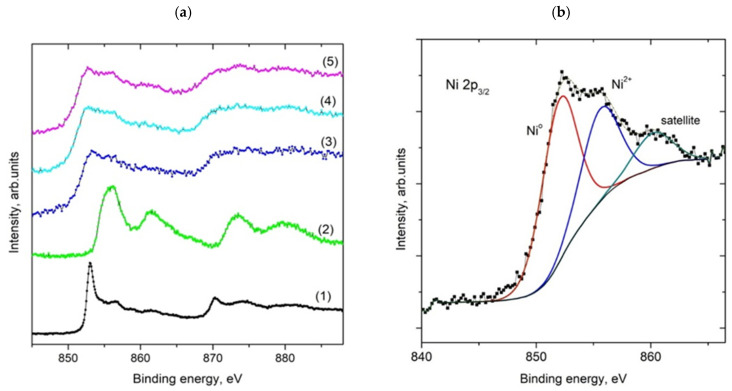
XPS of Ni 2p photoelectrons for Ni metal (1), Ni oxide (2), Ni(a)* (3), NiO400(b)* (4), and Ni(OH)_2_(b)* (5) catalysts (**a**), and fitted high-resolution Ni 2p3/2 spectrum for the Ni(OH)_2_(b)* catalyst (**b**). The spectra of Ni 2p3/2 electrons for the other two catalysts are within the margin for error and are included in the supporting information.

Using X-ray photoelectron spectroscopy (XPS), three samples, Ni(a)*, Ni(OH)_2_(b)*, and NiO400(b)*, were examined after catalysis. The wide-scan spectra (Figure S6), normalized to the same height and vertically shifted for clarity, show the presence of Ni, Si, O, and C only. In Figure 9a, the spectra of catalyst samples, together with those of metallic nickel and nickel oxide acquired in the Ni 2p region, are displayed. The fitted Ni 2p_3/2_ spectrum of the Ni(OH)_2_(b)* catalyst is shown with assignments of individual components in Figure 9b. The fitted and assigned spectra of Ni 2p_3/2_ photoelectrons of Ni(a)* and NiO400(b)* catalysts are displayed in the supporting material (Figure S7). The low-binding-energy component located at ~852.5 eV belongs to metallic Ni, while the higher-binding-energy component is consistent with the presence of Ni^2+^. Assuming a homogeneous sample, the elemental atomic concentrations displayed in Table 4 were calculated from integrated intensities of Ni 2p, Si 2p, O 1s, and C 1s spectra after their correction for pertinent photoionization cross sections and the transmission function of the spectrometer using CasaXPS software.

It should be mentioned that samples were, in fact, not homogeneous and were formed by bunches of catalytically active particles dispersed in larger particles of silica, as evident from SEM images (Figure 8). This inhomogeneity not only influenced the results of quantitative analysis but also resulted in broadening of the spectra, as can be seen from the spectra of C 1s photoelectrons (Figure S7d), where the dominant photoemission peak is associated with carbon atoms in C-C and C-H bonds and a high-binding-energy tail caused by the presence of oxygen-containing functionalities.

XPS analysis based on high-resolution spectra indicated that Ni(a)* has nearly the same carbon content on its surface as the two other samples. This is an interesting result as the duration of the experiment conducted with this catalyst was two times longer (42 vs. 21 h). Therefore, twice as much carbon dioxide passed through the sample, but the amount of carbon deposited was not twice as high as that for the other two samples. For the other two catalysts, XPS showed a significantly greater amount of carbon in Ni(OH)_2_(b)*, which may be due to its higher catalytic activity, which can cause higher deposition of carbon compared to NiO400(b)*. The high-resolution XPS of Ni 2p photoelectrons for the three post-catalytic samples is nearly identical. Their fitting did not show the differences in the populations of Ni oxidation states for the measured catalysts (Figure 9b). These spectra show a significant content (about 50%) of metallic Ni, which is essential for their excellent catalytic activity due to the activation of the hydrogen molecules for their reaction with CO_2_ molecules. However, the total amount of Ni on the surface of these three tested catalysts was significantly different, but due to the inhomogeneity of the measured samples (influence of SiO_2_ inert), these differences cannot be interpreted as a major argument for their catalytic activity. The influence of oxygen vacancies on catalytic activity is the most important parameter affecting the performance of metal oxide catalysts in heterogeneous catalysis [48]. However, in the case of the conducted catalytic experiments, it is difficult to evaluate their influence since a large part of the O 1s XPS (Figure S8a–c) came from the SiO_2_ added to the NiO_x_ catalysts before the catalytic experiments.

### 3.7. Results of Catalytic Hydrogenation of CO_2_ Using Different Ni-Based Catalysts

The hydrogenation of CO_2_ on Ni-based catalysts follows two possible pathways, one of which starts with the formation of CO via the reverse water gas shift reaction (RWGS) and its subsequent transformation to methane via the carbide pathway. The second pathway starts with the formate pathway, which then leads back to the carbide pathway of methane formation [49]. Metallic Ni plays a crucial role in these reactions because its surface is essential for activating hydrogen molecules for their subsequent reaction with CO_2_ [50]. Regardless of the starting form of Ni in the catalyst (metallic Ni or any oxide), the active form always contains metallic Ni, formed in the reducing reaction atmosphere, as demonstrated by the XPS of the spent catalysts.

The catalytic experiments’ main products were CH_4_ and CO, along with an almost immeasurable quantity of C_2_H_6_ (less than 0.1%) and C_3_H_8_ (less than 0.01%). Exemplary GC chromatograms of these products are presented in Figure S9a,b.

#### 3.7.1. Preliminary Catalytic Tests Using a Temperature Ramp

**Figure 10:**
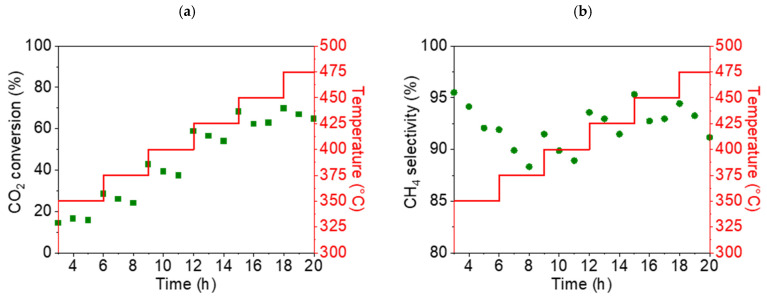
Conversion rate of CO_2_ (**a**) and selectivity for CH_4_ (**b**) for the NiO1000(a) sample (displayed as green points in both cases) during the temperature ramp from 350 up to 475 °C.

**Figure 11:**
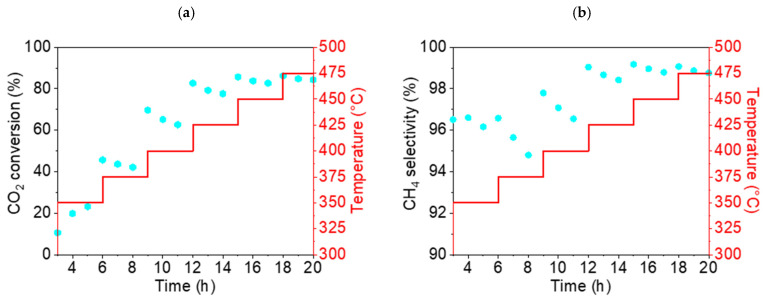
Conversion rate of CO_2_ (**a**) and selectivity for CH_4_ (**b**) for the NiO400(b) sample (displayed as blue points in both cases) during the temperature ramp from 350 up to 475 °C.

As can be seen in the graphs presented in Figure 10, the highest conversion rate of CO_2_ (68%) and the highest selectivity for CH_4_ (95.3%) were obtained for NiO1000(a) at a temperature of 450 °C. For the second sample, NiO400(b), the highest conversion rate of CO_2_ and the highest selectivity for CH_4_ were also found at 450 °C, at 86% and 99%, respectively (Figure 11).

Based on the results of these preliminary catalytic tests, a reaction temperature of 450 °C was identified as being optimal for all subsequent catalytic tests of stable methane production.

#### 3.7.2. Catalytic Efficiency of Catalysts with a Ni(a) Precursor (a-Group Catalysts)

**Figure 12:**
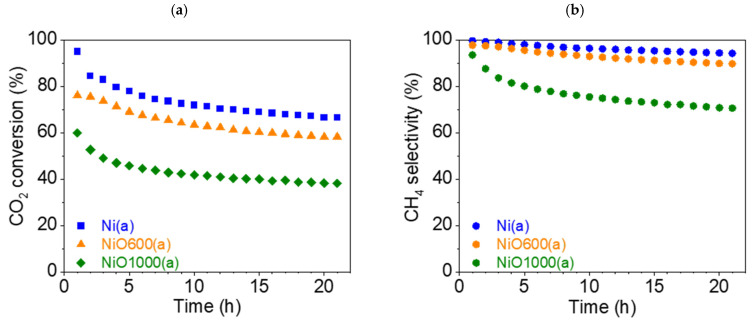
(**a**) Conversion rate of CO_2_ and (**b**) CH_4_ selectivity (●) of a-group catalysts at 450 °C: Ni(a) (■, blue), NiO600(a) (▲, orange), and NiO1000(a) (◆, green).

This section discusses the catalytic properties of nickel-derived catalysts (a-group catalysts): Ni(a), NiO600(a), and NiO1000(a). The CO_2_ conversion rate X_CO2_ dropped significantly for all three samples during the experiment. It is noteworthy that in the case of Ni(a) and NiO1000(a), there was a significant drop in the first hour of the test, from 94.9 (t = 1 h) to 84.3% (t = 2 h) and from 59.9 (t = 1 h) to 52.6% (t = 2 h), respectively (Figure 12a). For NiO600(a), the decrease in the conversion rate during the first hour of the experiment was less than 1%, from 76.0 (t = 1 h) to 75.5% (t = 2 h). However, Ni(a) demonstrated the best conversion rate throughout the entire 21 h test: the maximum was 94.9% (t = 1 h) and the minimum was 66.4% (t = 21 h). Therefore, the overall decrease in conversion rate during the 21 h test was 28.5%. The oxides showed significantly worse conversion rates overall, but the relative decrease was less than that of nickel Ni(a), from 76.0 (t = 1 h) to 58.2% (t = 21 h) (decrease 17.8%) for NiO600(a) and from 59.9 (t = 1 h) to 38.2% (t = 21 h) (decrease 21.7%) for NiO1000(a).

The selectivity S_CH4_ of all samples also fell. Additionally, the absolute values varied significantly compared to the conversion rate (Figure 12b). S_CH4_ for Ni(a) was close to 100% at the beginning of the test (99.6%, t = 1 h) and slightly decreased to 94.2% at the end of the test (t = 21 h). S_CH4_ for NiO600(a) also fell by less than 10%, from 97.7 (t = 1 h) to 89.7% (t = 21 h). However, in the case of NiO1000(a), the decrease in S_CH4_ was more significant—from 93.5 (t = 1 h) to 70.6% (t = 21 h). Moreover, in the first third of the test, the drop roughly corresponded to an exponential decrease, after which it became linear.

The maximum selectivity S_CO_ (Figure S10a) for the undesirable product, CO, was less than 6% (t = 21 h) for Ni(a) and less than 11% (t = 21 h) for NiO600(a). However, in the case of NiO1000(a), the increase in CO selectivity became very significant—from 6.5 (t = 1 h) to 29.4% (t = 21 h). The graphs of the reaction yield η_CH4_ and space–time yield STY_CH4_ can be found in the Supplementary Materials (Figures S11a and S12a).

Summing up the results obtained with this group of catalysts, oxidation and increases in temperature during the thermal treatment of precursors significantly worsened the catalytic properties of Ni(a) MPs. This suggests that the initially prepared pure nickel precursor Ni(a) was a better catalyst than oxides prepared through thermal treatment above its oxidation temperature. Subsequent hydrogen activation of the oxides at the start of the catalytic experiment was not sufficient to obtain high and stable catalytic activity compared with metallic Ni. Additionally, using temperature treatment at very high temperatures can worsen the catalytic activity of the final catalyst due to the recrystallization of primary crystals, forming nonactive crystal facets [51].

#### 3.7.3. Catalytic Efficiency of Catalysts with a Ni(OH)_2_(b) Precursor (b-Group Catalysts)

This section discusses the catalytic properties of nickel hydroxide-derived catalysts (b-group catalysts): Ni(OH)_2_(b), NiO400(b), and NiO600(b). Among these, the precursor nickel hydroxide Ni(OH)_2_(b) was converted entirely to NiO not only by oxidative thermal treatment but also by the temperature used for the catalytic reaction experiment (450 °C), as the temperature for oxidative transformation was observed to be below 400 °C (Figure 2). On the other hand, it should be remembered that during the activation phase, the reduction of oxides to metallic Ni occurs.

**Figure 13:**
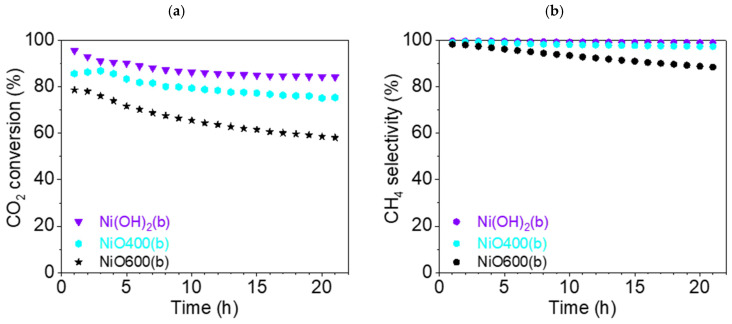
(**a**) Conversion of CO_2_ and (**b**) CH_4_ selectivity (●) of b-group catalysts at 450 °C: Ni(OH)2(b) (▼, violet), NiO400(b) (⬢, light blue), and NiO600(b) (★, black).

The CO_2_ conversion rate X_CO2_ dropped for all samples during the 21 h test. For Ni(OH)_2_(b) and NiO400(b), it fell by just over 10%—from 95.6% (t = 1 h) to 84.2% (t = 21 h) and from 86.9% (t = 3 h) to 75.4% (t = 21 h), respectively (Figure 13). During the experiment, the X_CO2_ for Ni(OH)_2_(b) was about 10% higher than that for NiO400(b), which is very significant for such similar catalysts. The third catalyst, NiO600(b), demonstrated a significantly worse conversion rate during catalysis, reducing from 78.6% (t = 1 h) to 58.1% (t = 21 h). Overall, all three catalysts showed an excellent conversion rate compared to other studies that used pure Ni-based catalysts [38].

Excellent selectivity for methane S_CH4_ was found for Ni(OH)_2_(b), at more than 99% during all 21 h experiments. Slightly inferior results were demonstrated for NiO400(b), at more than 97% during the test. However, the third catalyst was significantly worse, as S_CH4_ decreased from 98.3 (t = 1 h) to 88.5% (t = 21 h).

The CO selectivity (Figure S10b) for Ni(OH)_2_(b) was less than 1%, and for NiO400(b), it was less than 3% during the entire catalysis testing period. For NiO600(b), the selectivity for CO grew significantly, from 1.7 (t = 1 h) to 11.5% (t = 21 h). The graphs of the reaction yield η_CH4_ and space–time yield STY_CH4_ can be found in the Supplementary Materials (Figures S11b and S12b).

It can also be argued that, in this case, the thermal treatment at a temperature much higher than the transformation temperature observed during the TG/DSC experiment worsened the properties of the catalyst. On the other hand, using a temperature close to the temperature required for the hydroxide’s transformation to an oxide did not significantly influence the catalytic efficiency of the precatalyst in comparison with the direct use of the hydroxide precursor. However, the transformation proceeding directly in the reaction conditions seems to be the best way to prepare a highly efficient catalyst.

#### 3.7.4. Catalytic Efficiency of Catalysts with a Ni(OH)_x_(c) Precursor (c-Group Catalysts)

**Figure 14:**
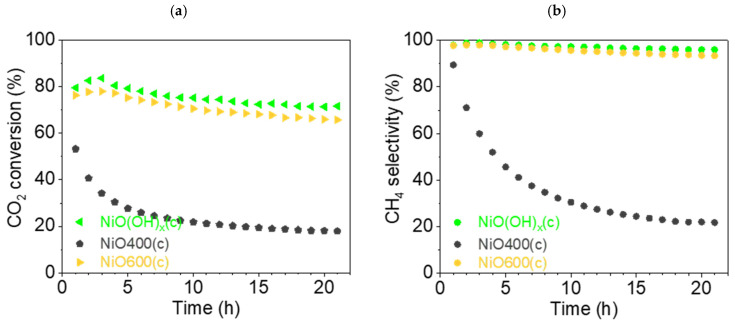
(**a**) Conversion of CO_2_ and (**b**) CH_4_ selectivity (●) of c-group catalysts at 450 °C: NiO(OH)_x_(c) (◄, light-green), NiO400(c) (⬟, gray), and NiO600(c) (▶, orange).

This section discusses the catalytic properties of nickel metahydroxide-derived catalysts (c-group catalysts): NiO(OH)_x_(c), NiO400(c), NiO600(c). In this case, as in previous cases, the primary form of the tested catalysts was nickel oxide. The precursor nickel metahydroxide NiO(OH)_x_(c) was completely converted to NiO at 450 °C, as can be seen from TG/DSC analysis (Figure 3). Using thermal treatment in an oxidative atmosphere, two other catalysts were prepared, NiO400(c) and NiO600(c). The trends observed in the case of the Ni(OH)_2_(b) precursor are different to the results observed in this section. Firstly, the catalytic activity of the prepared catalysts was not as good as that observed for b-group catalysts. The CO_2_ conversion rate X_CO2_ decreased during catalysis, but did not decrease in the same manner for the tested samples. For NiO(OH)_x_(c) and NiO600(c), the drop in conversion rate was moderate: from 83.6 (t = 3 h) to 71.4% (t = 21 h) and from 78.0 (t = 3 h) to 65.7% (t = 21 h), respectively. It is noteworthy that in both cases, the maximum value was not observed in the first hour of the experiment but in the third, as can be seen on the graphs (Figure 14). Remarkably, NiO400(c), which was obtained at a lower calcination temperature than NiO600(c), had a much poorer conversion rate, dropping from 53.3 (t = 1 h) to 17.9% (t = 21 h), representing the worst values for all the catalysts tested in this study.

The values of CH_4_ selectivity S_CH4_ varied according to similar patterns, as was observed for the conversion rate. For metahydroxide NiO(OH)_x_(c), the peak value was 98.9 (t = 3 h), dropping to 95.9% (t = 21 h), while for NiO600(c), the peak value was 98.0 (t = 3 h), falling to 93.5% (t = 21 h). Meanwhile, NiO400(c) showed significantly worse selectivity, with the graph showing a hyperbolic 4-fold drop from 89.4 (t = 1 h) to 21.8% (t = 21 h).

The CO selectivity (Figure S10c) for NiO(OH)_x_(c) increased from 1.1 (t = 3 h) to 4.0% (t = 21 h) and for NiO600(c) from 2.1 (t = 3 h) to 6.6% (t = 21 h) during catalysis. For NiO400(c), selectivity for CO production grew greatly, by more than 7 times, from 10.6 (t = 1 h) to 78.2% (t = 21 h).

The graphs of the reaction yield η_CH4_ and space–time yield STY_CH4_ can be found in the Supplementary Materials (Figures S11c and S12c).

Summarizing the results for c-group catalysts, it can be argued that NiO(OH)_x_(c) and NiO600(c) displayed methanation from the point of view of selectivity and conversion, similar to most catalysts in the other two groups. However, the abnormally low catalytic activity of NiO400(c) is surprising. The large drop in selectivity for CH_4_ and the conversion rate of CO_2_ with the same increase in selectivity for CO demonstrates a principal change in the catalytic properties of all the Ni-based catalysts tested here. A possible reason for this is the irregular, rough structure of the sample, which was seen in the SEM images. The second reason for such a different catalytic activity could be connected with the imperfect transformation of non-stochiometric hydroxide into an active NiO catalyst. However, to elucidate the real reasons for the existence of this phenomenon, a more detailed study of the changes in the chemistry of this catalyst should be optimally supported by theoretical calculations and in situ measurements.

#### 3.7.5. Stability Tests of Ni-Based Catalysts

**Figure 15:**
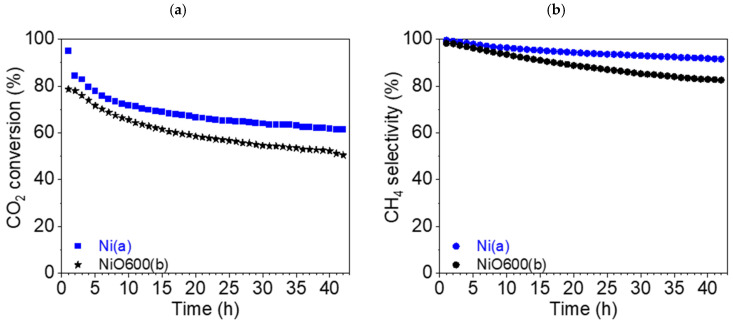
(**a**) Conversion of CO_2_ and (**b**) CH_4_ selectivity (●) of Ni(a) (■, blue) and NiO600(b) (★, black) at 450 °C during a stability test.

Stability tests (42 h) were carried out for two catalysts: Ni(a) and NiO600(b). In the case of Ni(a), the CO_2_ conversion rate X_CO2_ fell from 94.9 (t = 1 h) to 61.4% (t = 42 h), i.e., the drop was nearly 35% (Figure 15a). The drop in selectivity for CH_4_ was not as substantial, being about 8.1%, from 99.6 (t = 1 h) to 91.5% (t = 42 h), as can be seen from Figure 15b.

The absolute values for the catalyst NiO600(b) look similar. The CO_2_ conversion rate X_CO2_ also decreased by about 25%: from 78.6 (t = 1 h) to 50.5% (t = 42 h) (Figure 15a). The decrease in selectivity for CH_4_ was more pronounced in this case, at approximately 16%, from 98.3 (t = 1 h) to 82.5% (t = 42 h) (Figure 15b).

The graphs of selectivity for CO production (S_CO_), reaction yield η_CH4_, and space–time yield STY_CH4_ can be found in the Supplementary Materials (Figures S10d, S11d, and S12d).

To summarize the stability tests, it can be deduced that in long-term catalysis, the metallic nickel Ni(a) precursor appeared to be a more stable catalyst in comparison with the oxide NiO600(b) prepared at elevated temperatures from Ni(OH)_2_(b).

### 3.8. Summary of Catalytic Results

In general, most of the tested samples of Ni-based catalysts showed high catalytic activity, with their basic parameters reaching thermodynamic limits at the reaction temperature, namely conversion rate of CO_2_ of 89.9% and a selectivity for CH_4_ production of 99.9% [52]. The degree of conversion was influenced by the physical state of the catalyst (specific surface area) and simultaneously by the chemical composition of precatalysts (see Table 5). As metallic Ni is the most efficient form of Ni-based catalyst for CO_2_ methanation, the catalyst Ni(a) showed the second highest starting conversion rate, regardless of its very low specific surface area. On the other hand, a high specific surface area was not a guarantee for high catalytic activity, as the precatalyst NiO(OH)_x_(c) with the second highest specific surface area value only ranked fourth regarding its starting conversion rate in the methanation reaction. This may be due to the fact that this compound forms relatively large, strongly agglomerated aggregates of needle-like crystals that protrude out from the surface of the aggregates and thus increase the value of the specific surface area. However, at elevated temperatures of several hundred degrees Celsius, these needles with diameters in the tens of nanometers at most disappeared from the surface of the aggregates (Figure S3h), which is likely the reason for the low catalytic activity at the optimum reaction temperature of 450 °C. Therefore, we can conclude that the effect of surface area on the catalytic activity is not as important as the presence of the well-defined form of Ni needed for optimal transformation to metallic Ni in the reaction atmosphere. The morphology of the precatalyst particles also plays an important role. Therefore, the best catalyst in the presented study was well-defined Ni(OH)_2_(b), which can be easily thermally transformed into the active catalyst in the reduction atmosphere, as can be deduced from TPR-H_2_ measurement (see Figure 6). Additionally, TPD-CO_2_ measurement confirmed excellent interaction with CO_2_ for this precatalyst. The positive collaboration of the high surface area and the presence of the metallic Ni formed in the reaction atmosphere (see XPS spectrum in Figure 9b) resulted in this compound having the best catalytic activity among all of the tested catalysts in this study. On the other hand, the high surface area was advantageous for stabilizing the catalytic efficiency of the studied catalysts. The best catalyst with the highest surface area in this study was also the catalyst whose performance did not change significantly during the standard experiment (21 h). However, the low surface area value of the pure Ni(a) precatalyst caused low resistance to deterioration of its catalytic efficiency, despite its high starting catalytic activity. The main reason for the significant decrease in the CO_2_ conversion rate was connected with the deposition of elemental carbon on its surface (see Table 4), preferably in the amorphous form, as was determined by Raman spectroscopy. Figure S13 shows the Raman spectrum (baseline subtracted and smoothed by Savizky–Golay algorithm) of Ni(OH)_2_(b), which contains two main broad bands with maxima at 1310 cm^−1^ (FWHM 82.39 cm^−1^) and 1593 cm^−1^ (FWHM 53.01 cm^−1^) of Raman shift, which indicates the presence of amorphous carbon or a carbon film, with these bands being correlated with the D and G bands of carbon materials, respectively [53]. Although the amount of deposited carbon determined by XPS was nearly the same for all three studied samples, the ratio of carbon content vs. specific surface area was highest for the Ni(a) catalyst, at 0.79, which was ten times higher in comparison with the value of 0.074 for the Ni(OH)_2_(b) catalyst. Therefore, carbon distribution on the surface of the Ni(a) catalyst with a low surface area was more limited, and commonly, the deterioration of the catalytic activity of catalysts with a low specific surface area, as is the case of the Ni(a) catalyst, was much higher in comparison with catalysts with a higher specific surface area, as seen for Ni(OH)_2_(b) (more than 10 times, see Table 5). To confirm this hypothesis, the ratio of the initial to the final CO_2_ conversion rate for the 21 h experiments can be used. In the case of the Ni(a) precatalyst, this ratio was 0.7, i.e., a decrease of 30%. In the case of the Ni(OH)_2_(b) precatalyst, this ratio was 0.88, i.e., a decrease of only 12%. Comparison of these relative values shows that the decrease in catalytic activity was 2.5 times higher for Ni(a) than for Ni(OH)_2_(b). Thus, these values qualitatively support the hypothesis of a decrease in catalytic activity due to the formation of surface deposits of reduced carbon.

**Figure 16:**
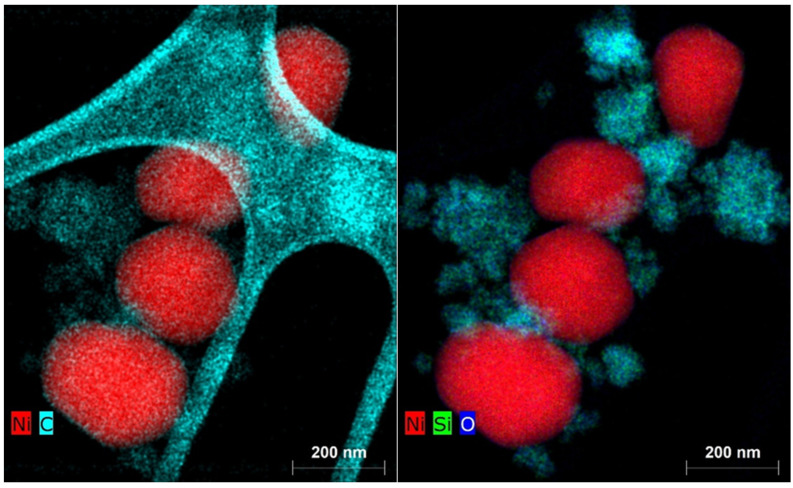
Elemental mapping of the main elements in the Ni(a) spent catalyst.

**Figure 17:**
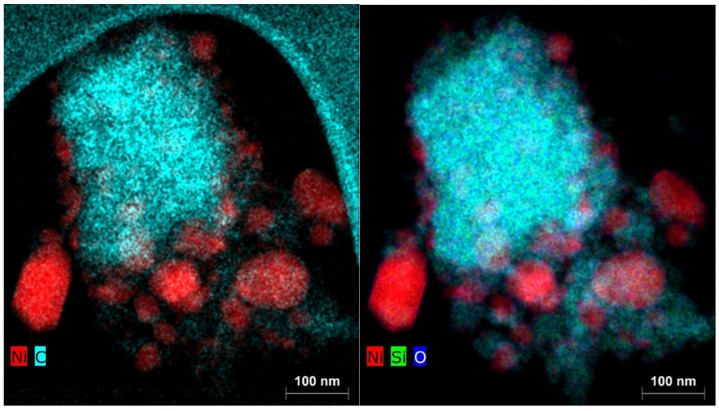
Elemental mapping of the main elements in the Ni(OH)_2_(b) spent catalyst.

The fact that the catalytic activity due to the deposition of elemental carbon during the catalytic reaction with the Ni(a) catalyst did not decrease in proportion to its smaller surface area compared to the Ni(OH)_2_(b) catalyst is due to the fact that carbon was deposited not only on the catalyst’s surface but also on the silica surface used as the antisintering agent. This fact is clearly documented by the EDX elemental mapping of the two catalysts in Figure 16 for Ni(a) and Figure 17 for Ni(OH)_2_(b).

Another important finding from the conducted experiments relates to the effect of heat treatment of the precatalysts. In all three cases (a–c), the most effective catalyst was the primary prepared substance, and its thermal treatment in an oxidizing atmosphere reduced the specific surface area; in connection with this fact, deterioration of the catalytic activity of the final precatalysts was observed.

A comparison of the key characteristics, selectivity for CH_4_, and conversion rate of CO_2_ with previously published research results is presented in Table 6. These studies showed the favorability of the use of the solvothermal method for the preparation of Ni-based methanation catalysts in comparison with the conventional precipitation method [54,55,56,57].

## 4. Conclusions

The experiments conducted in this study demonstrated that the solvothermal synthesis of Ni-based catalysts for CO_2_ methanation is an efficient way to prepare highly active catalysts. The obtained results of catalytic activity showed that the final formation of the active catalyst at elevated temperatures from precursors in a reducing reaction atmosphere directly in the reactor could be preferred over thermal transformation at elevated temperatures in air. The results showed that there are two main factors influencing the catalytic efficiency of the final catalysts, the high specific surface area and metallic Ni (preferably formed during the activation period in the reactor), which are needed for optimal catalysis. However, stabilizing Ni-based catalysts by anchoring them onto suitable supports (typically oxides of less noble metals) will be required to improve the time stability of the catalytic activity of the tested pure Ni-based catalysts, as was proven by other studies published previously. Furthermore, optimization of the catalytic activity in order to lower the optimal reaction temperature via decoration of the Ni catalytic particles with noble metals (e.g., Pt, Rh, Pd, Ru) or rare earth metals (e.g., Y, La, Eu) will be another direction of subsequent research.

## Figures and Tables

**Table 1:** Summary table of synthesis of Ni-based samples.

Product	Solution	m (NaAc·3H_2_O), g	Calcination, Sample + T, °C *
Ni(a)	80 mL EG	11.92	No
NiO600(a)	--//--	--//--	Yes, Ni(a), 600 °C
NiO1000(a)	--//--	--//--	Yes, Ni(a), 1000 °C
Ni(OH)_2_(b)	40 mL EG + 40 mL Et	11.92	No
NiO400(b)	--//--	--//--	Yes, Ni(OH)_2_, 400 °C
NiO600(b)	--//--	--//--	Yes, Ni(OH)_2_, 600 °C
NiO(OH)_x_(c)	40 mL EG + 40 mL Et	5.96	No
NiO400(c)	--//--	--//--	Yes, NiO(OH)_x_, 400 °C
NiO600(c)	--//--	--//--	Yes, NiO(OH)_x_, 600 °C

* All calcination temperatures were selected based on thermogravimetric diagrams.

**Table 2:** Summary table of XRD results—quantification of crystalline phase composition from Rietveld refinement of XRD patterns.

Catalyst	XRD Measurement, Stoichiometry			
	Ni	NiO	α-Ni(OH)_2_	(Ni(OH)_2_(NiOOH)_0.167_)_0.857_
Ni(a)	100% (cubic) ^1^			
NiO600(a)		100%		
NiO1000(a)		100%		
Ni(OH)_2_(b)			100% ^2^	
NiO400(b)	0.8%	99.2%		
NiO600(b)		100%		
NiO(OH)_x_(c)				100%
NiO400(c)	0.8%	99.2%		
NiO600(c)		100%		

^1^ Trace Ni (hexagonal); ^2^ trace Ni.

**Table 3:** Surface area, pore size, and total volume of Ni-based samples.

Sample Series	(a)			(b)			(c)		
Sample Name	Ni	NiO600	NiO1000	Ni(OH)_2_	NiO400	NiO600	NiO(OH)_x_	NiO400	NiO600
BET surface area, m^2^/g	7.03	4.51	1.09	83.99	54.41	13.79	70.07	28.56	12.34
Pore volume, cm^3^/g	0.0154	0.0070	0.0012	0.1090	0.0736	0.0166	0.1157	0.0369	0.0125
Average pore width, nm	10.1	6.8	4.1	5.6	5.8	4.5	6.4	5.6	4.0

**Table 4:** Populations of elements (in atomic%) on the catalyst’s surface after catalysis evaluated from XPS measurements.

Catalyst	Element			
	Ni	Si	O	C
Ni(a)*	1.0	30.8	62.7	5.5
NiO400(b)*	2.4	30.1	62.7	4.8
Ni(OH)_2_(b)*	1.9	30.2	61.7	6.2

**Table 5:** Comparison of the specific surface area (Ssp) and catalytic activity (CO_2_ conversion at start X_CO2,start_, and at the end X_CO2,end_ of the catalytic experiment) of the studied Ni-based catalysts.

Sample	S_sp_, m^2^/g	X_CO2,start_	X_CO2,end_	S_CH4,start_	S_CH4,end_	Rank, X_CO2,start_	Rank, X_CO2,end_	Rank, S_sp_
Ni(OH)_2_(b)	84.0	95.6	84.2	99.8	99.0	1.	1.	1.
Ni(a)	7.0	94.9	66.4	99.6	94.2	2.	4.	7.
NiO400(b)	54.4	86.9	75.1	99.4	97.4	3.	2.	3.
NiO(OH)_x_(c)	70.1	83.6	71.4	98.9	96.0	4.	3.	2.
NiO600(b)	13.8	78.6	58.1	98.3	88.5	5.	7.	5.
NiO600(c)	12.3	78.0	65.7	97.7	93.4	6.	5.	6.
NiO600(a)	4.5	76.0	58.2	97.7	89.7	7.	6.	8.
NiO1000(a)	1.1	59.9	38.2	93.5	70.6	8.	8.	9.
NiO400(c)	28.6	53.3	17.9	89.4	21.8	9.	9.	4.

**Table 6:** Comparison of the catalytic activity (average CO_2_ conversion rate and CH_4_ selectivity) of the studied Ni-based catalysts with some previously published cases.

Sample	X_CO2(average)_	S_CH4(average)_	T (°C)	Source
Ni(a)	80.4	96.9	450	this study
Ni(OH)_2_(b)	89.9	99.4	450	this study
NiO400(b)	81.0	98.4	450	this study
Ni/Al_2_O_3_	88	97	450	[54]
Ce-Ni/SA	80	96	450	[55]
NiO/CaO·2Al_2_O_3_	79.1	98.1	450	[56]
12Ni_3_Fe	84.3	100.0	420	[57]

## Supplementary Materials

The following supporting information can be downloaded at https://www.mdpi.com/article/10.3390/nano15171379/s1, Figure S1: XRD characterization of Ni-consisted catalysts: (a) Ni(a), (b) NiO600(a), (c) NiO1000(a), (d) NiO400(b), (e) NiO600(b), (f) NiO(OH)_x_(c), (g) NiO400(c) and (h) NiO600(c); Figure S2: Dynamic XRD study of NiO sample reduction under H_2_ atmosphere: (a) NiO600(b), (b) NiO600(c); Figure S3: TEM characterization of Ni-consisted samples: (a) Ni(a), (b) NiO600(a), (c) NiO1000(a), (d) Ni(OH)2(b) (e) NiO400(b), (f) NiO600(b), (g) NiO(OH)_x_(c), (h) NiO400(c) and (i) NiO600(c); Figure S4: EDX analysis of precatalysts (a) Ni(a), (b) Ni(OH)_2_(b), and (c) NiO400(c); Figure S5: Isotherm linear plots and BET surface area plots for (a) Ni(a) and (b) NiO600(a); Figure S6: X-ray photoelectron spectroscopy (XPS) survey spectra: Ni(a)* (1, blue), NiO400(b)* (2, light-blue), and Ni(OH)_2_(b)* (3, pink); Figure S7: Fitted spectra of Ni 2pš/2 photoelectrons of (a) Ni(a)* and (b) NiO400(b)* catalyst samples; Figure S8: Fitted spectra of O 1s photoelectrons of catalyst samples of (a) Ni(a)*, (b) NiO400(b)*, and (c) Ni(OH)_2_(b)* catalysts, and (d) of C 1s photoelectrons of Ni(a)* (1, blue), NiO400(b)* (2, light-blue), and Ni(OH)_2_(b)* (3, pink). Spectra are normalized to the same height and vertically shifted for the sake of clarity; Figure S9: GC chromatograms taken during the catalytic experiments with Ni-consisted catalysts (10th hour): (a) Ni(a), (b) NiO600(a), (c) NiO1000(a), (d) Ni(OH)_2_(b), (e) NiO400(b), (f) NiO600(b), (g) NiO(OH)_x_(c), (h) NiO400(c), and (i) NiO600(c); Figure S10: Selectivity CO for a-group (a), b-group (b), c-group (c) of catalysts and in stability test (d); Figure S11: Reaction yield for a-group (a), b-group (b), c-group (c) of catalysts and in stability test (d); Figure S12: Space-time yield for a-group (a), b-group (b), c-group (c) of catalysts and in stability test (d); Figure S13: Raman spectrum of Ni(OH)2(b) sample, 780 nm, 10 mW.
