# Supplementary material for: The Solvothermal Method: An Efficient Tool for the Preparation of Ni-Based Catalysts with High Activity in CO2 Methanation
Source: Nanomaterials (Basel). 2025 Sep 6;15(17):1379. doi: 10.3390/nano15171379 (PMC12430002; doi:10.3390/nano15171379)
Supplement: Supplementary file 1 [file nanomaterials-15-01379-s001.zip › nanomaterials-3804087-supplementary.pdf]

Simple solvothermal synthesis of Ni based catalysts for modified Fischer-Tropsch reaction.

Arkadii Bikbashev [1], Tomáš Stryšovský [1], Martina Kajabová [1], Zuzana Kovářová [1], Arati Prakash Tibe [1], Karolína Simkovičová [1,2], Robert Prucek [1], Aleš Panáček [1], Josef Kašlík [3], Patrizia Frontera [4], Kouřil Roman [5], Arian Grainca [6], Carlo Pirola [6], Libor Brabec [7], Zdeněk Bastl [8], Štefan Vajda [2] and Libor Kvítek [1,*]

[1] Department of Physical Chemistry, Faculty of Science, Palacký University Olomouc, 17. listopadu 12, 77146 Olomouc, Czech Republic

[2] Department of Nanocatalysis, J. Heyrovský Institute of Physical Chemistry, Czech Academy of Sciences, Dolejskova 2155/3, 18223 Prague 8, Czech Republic

[3] Czech Advanced Technology & Research Institute CATRIN, Regional Centrum of Advanced Technologies & Materials, Palacký University Olomouc, Slechtitelu 27, 78371 Olomouc, Czech Republic

[4] Department of Civil, Energy, Environmental and Material Engineering, Mediterranea University of Reggio Calabria, Reggio Calabria, 89124, Italy

[5] Department of Biophysics, Faculty of Science, Palacký University Olomouc, Slechtitelu 27, 78371 Olomouc, Czech Republic

[6] Department of Chemistry, University of Milan, Via Golgi 19, 20133 Milano, Italy

[7] Center for Innovations in the Field of Nanomaterials and Nanotechnologies, J. Heyrovský Institute of Physical Chemistry, Czech Academy of Sciences, Dolejskova 2155/3, 18223 Prague 8, Czech Republic

8 Department of Low-dimensional Systems, J. Heyrovský Institute of Physical Chemistry, Czech Academy of Sciences, Dolejskova 2155/3, 18223 Prague 8, Czech Republic

* Correspondence: libor.kvitek@upol.cz; Tel.: + 420 585 634 420

a)

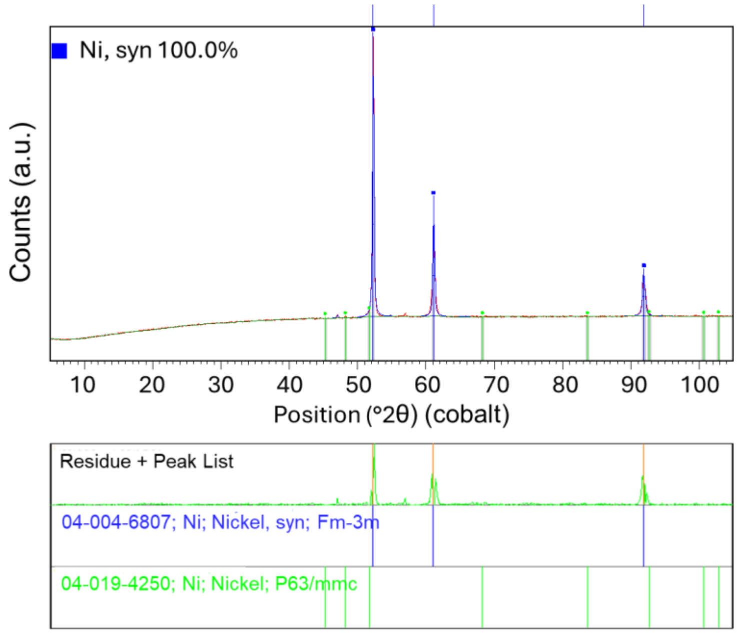

Ni, syn 100.0%
Counts (a.u.)
10
20
30
40
50
60
70
80
90
100
Position (°2θ) (cobalt)
Residue + Peak List
04-004-6807; Ni; Nickel, syn; Fm-3m
04-019-4250; Ni; Nickel; P63/mmc

b)

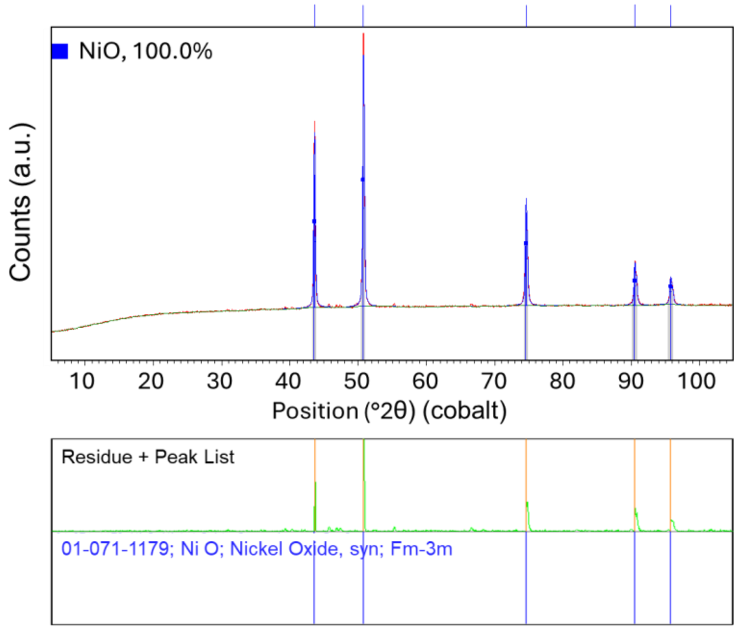

NiO, 100.0%
Counts (a.u.)
10
20
30
40
50
60
70
80
90
100
Position (°2θ) (cobalt)
Residue + Peak List
01-071-1179; Ni O; Nickel Oxide, syn; Fm-3m

c)

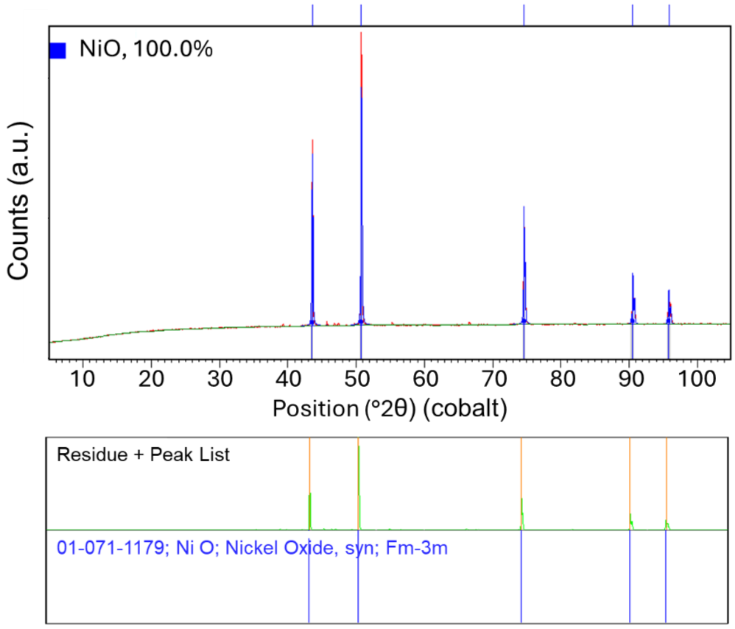
NiO, 100.0%
Counts (a.u.)
10 20 30 40 50 60 70 80 90 100
Position (°2θ) (cobalt)
Residue + Peak List
01-071-1179; Ni O; Nickel Oxide, syn; Fm-3m

d)

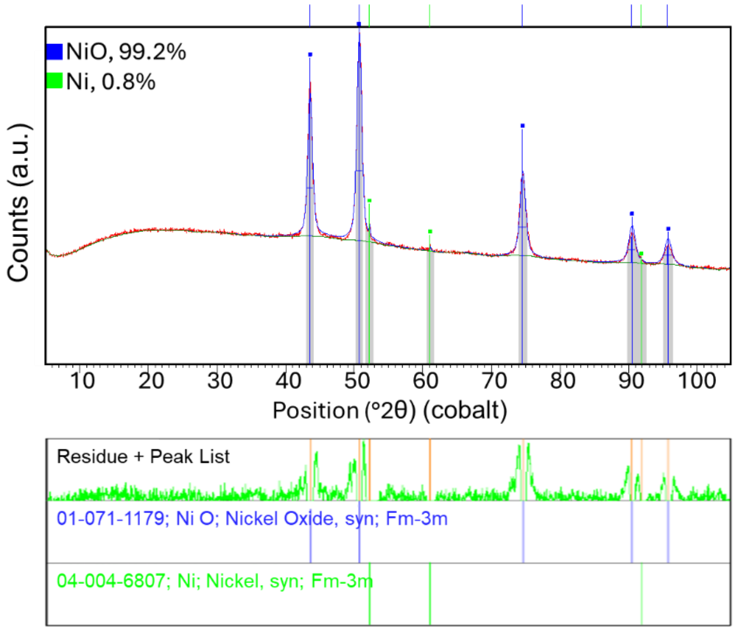
NiO, 99.2%
Ni, 0.8%
Counts (a.u.)
10 20 30 40 50 60 70 80 90 100
Position (°2θ) (cobalt)
Residue + Peak List
01-071-1179; Ni O; Nickel Oxide, syn; Fm-3m
04-004-6807; Ni; Nickel, syn; Fm-3m

e)

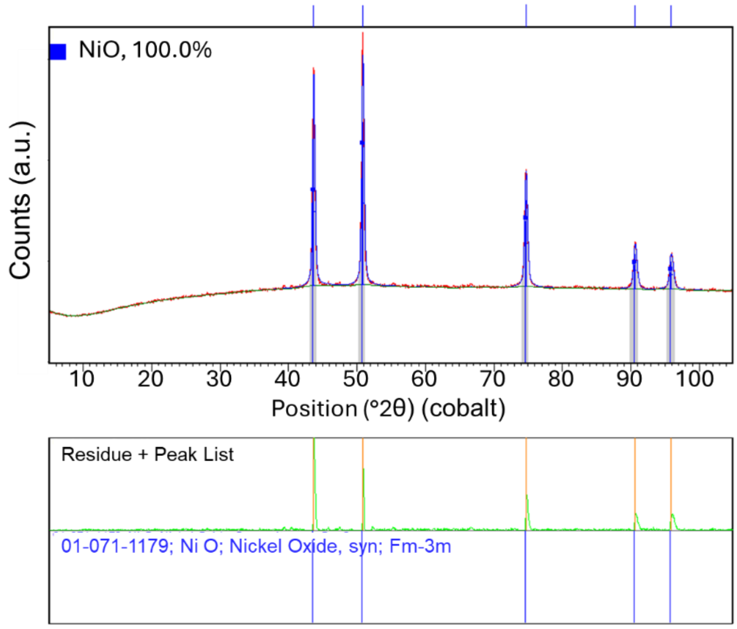
NiO, 100.0%
Counts (a.u.)
10 20 30 40 50 60 70 80 90 100
Position (°2θ) (cobalt)
Residue + Peak List
01-071-1179; Ni O; Nickel Oxide, syn; Fm-3m

f)

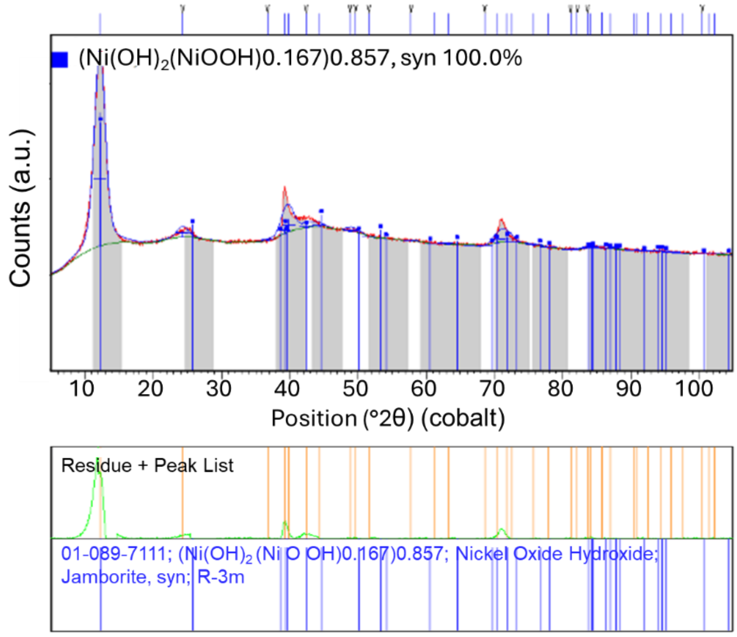
(Ni(OH)2(NiOOH)0.167)0.857, syn 100.0%
Counts (a.u.)
10 20 30 40 50 60 70 80 90 100
Position (°2θ) (cobalt)
Residue + Peak List
01-089-7111; (Ni(OH)2(Ni O OH)0.167)0.857; Nickel Oxide Hydroxide; Jamborite, syn; R-3m

g)

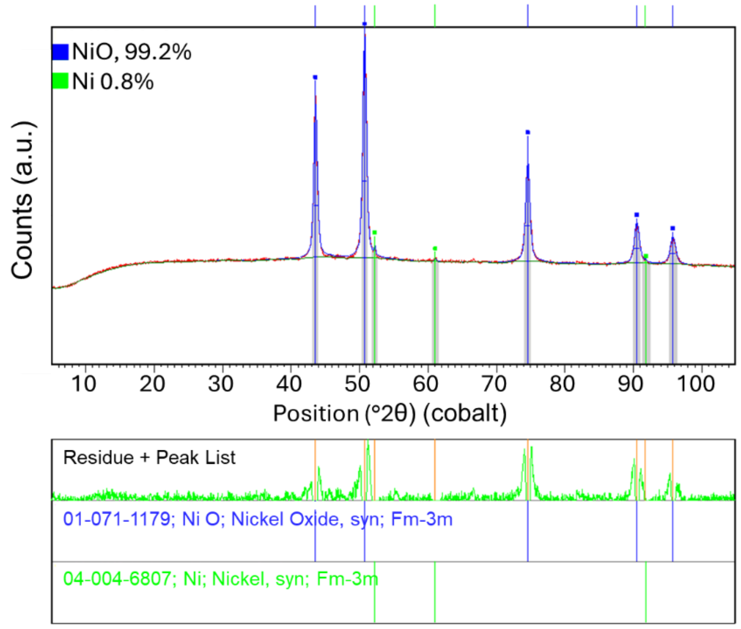

h)

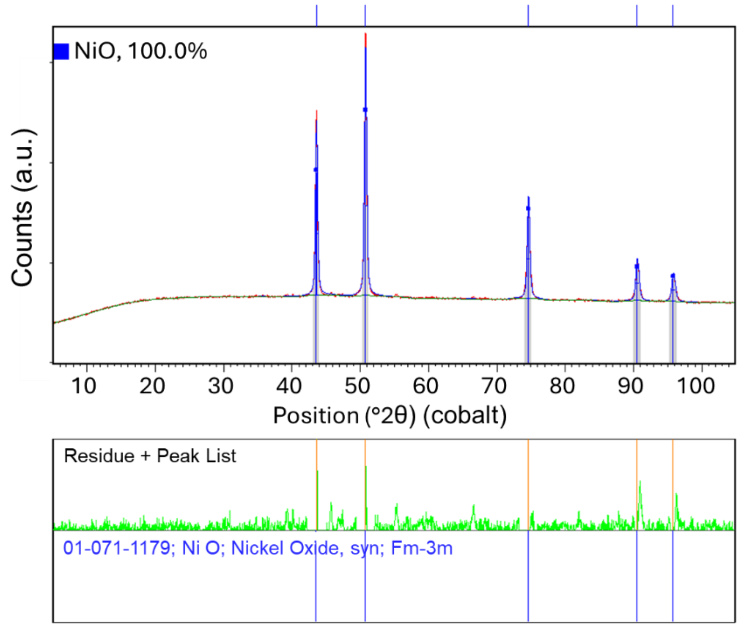

Figure S1. *XRD characterization of Ni-consisted catalysts: a) Ni(a), b) NiO600(a), c) NiO1000(a), d) NiO400(b), e) NiO600(b), f) NiO(OH)x(c), g) NiO400(c) and h) NiO600(c)*

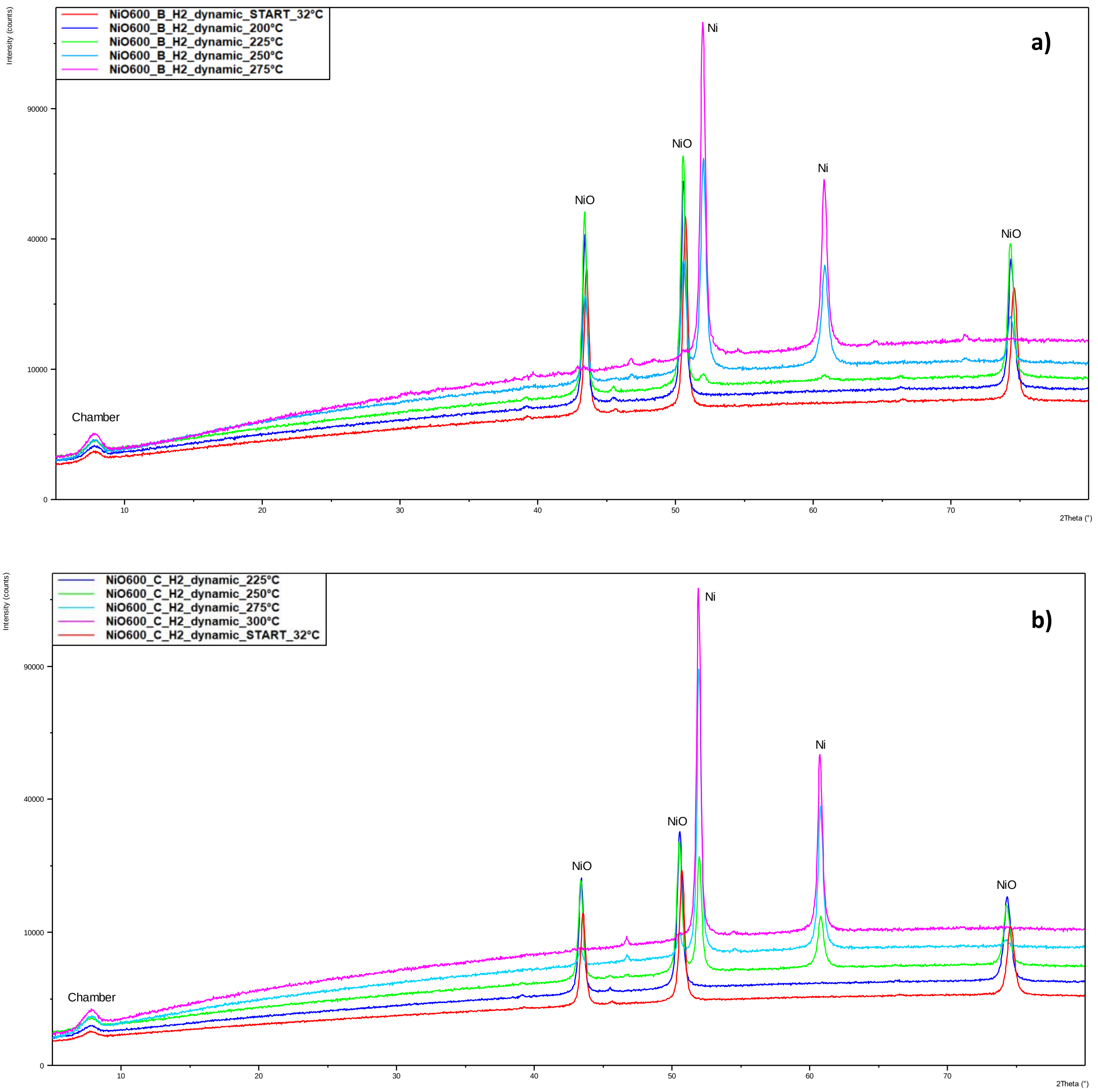

Figure S2. *Dynamic XRD study of NiO samples reduction under H_2 atmosphere: a) NiO600(b), b) NiO600(c).*

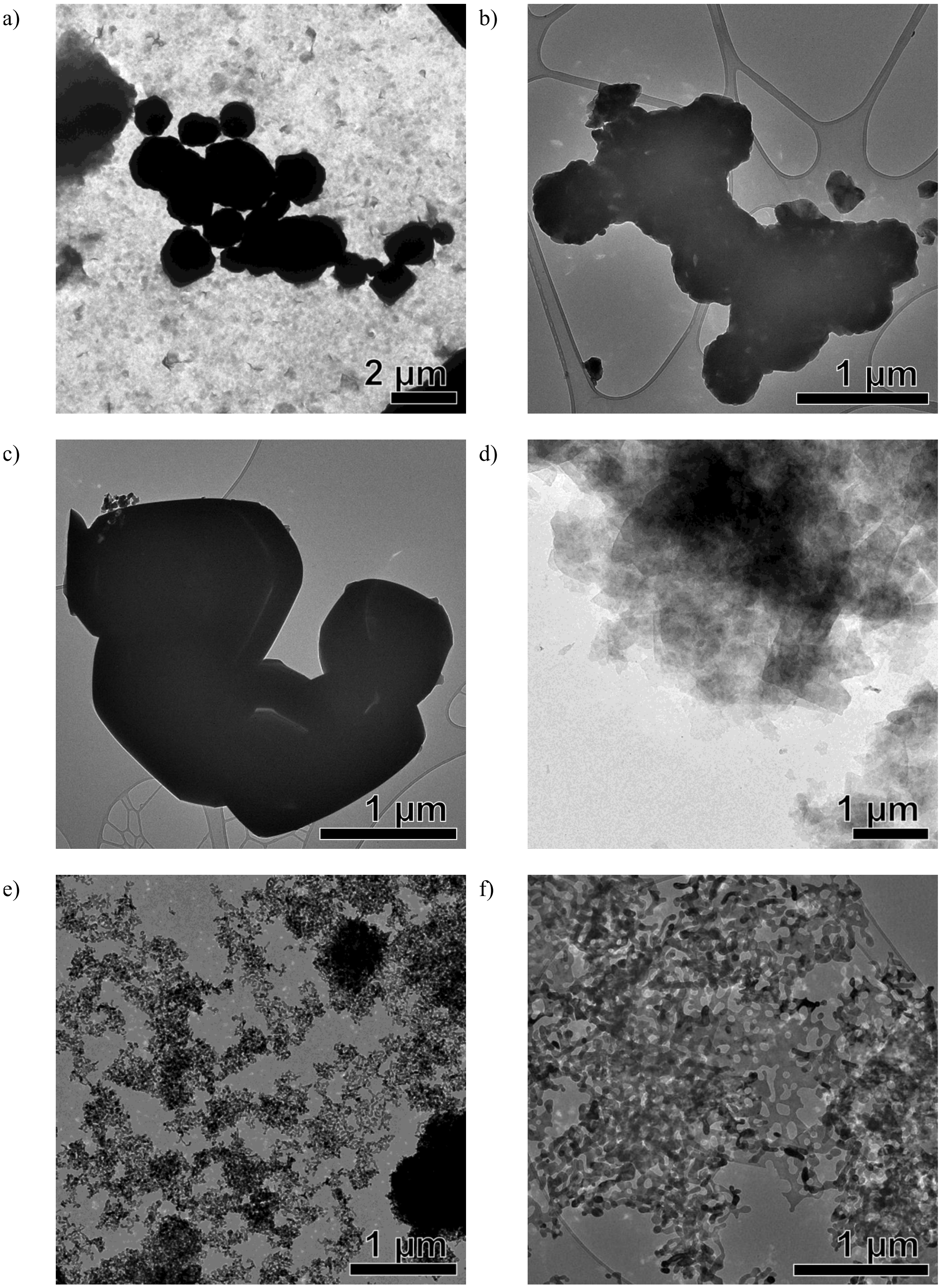
a)
2 µm
b)
1 µm
c)
1 µm
d)
1 µm
e)
1 µm
f)
1 µm

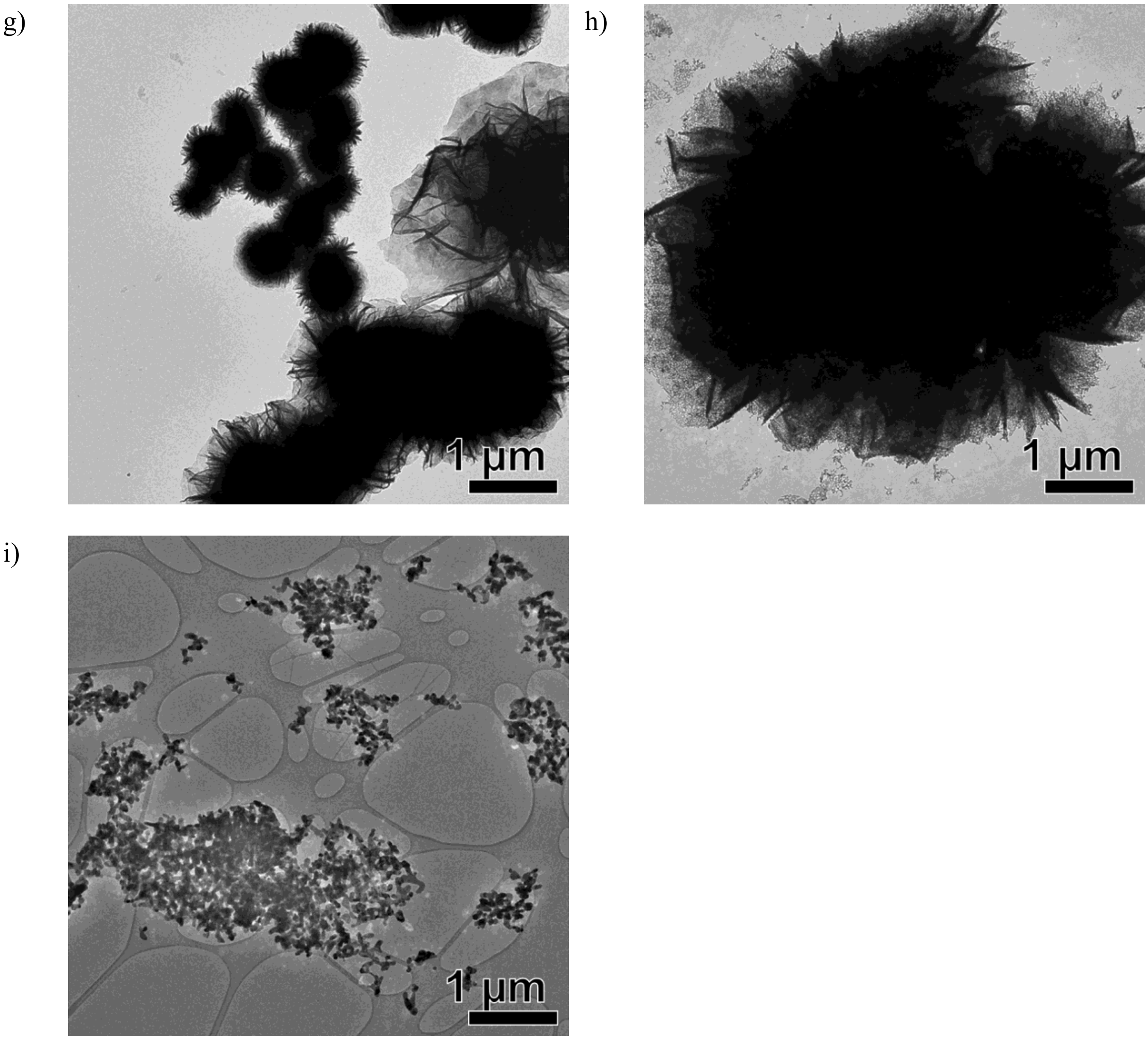

Figure S3. *TEM characterization of Ni-consisted samples: a) Ni(a), b) NiO600(a),* c) *NiO1000(a), d) $Ni(OH)_2$(b) e) NiO400(b), f) NiO600(b), g) $NiO(OH)_x$(c)***,** *h) NiO400(c) and i)* NiO600*(c)***.**

Ni(a)

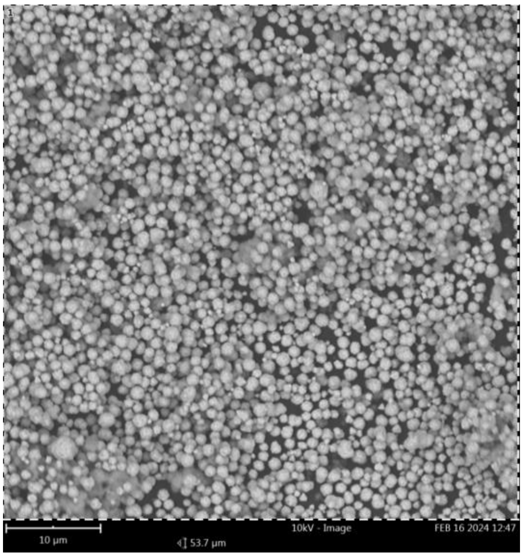

a)

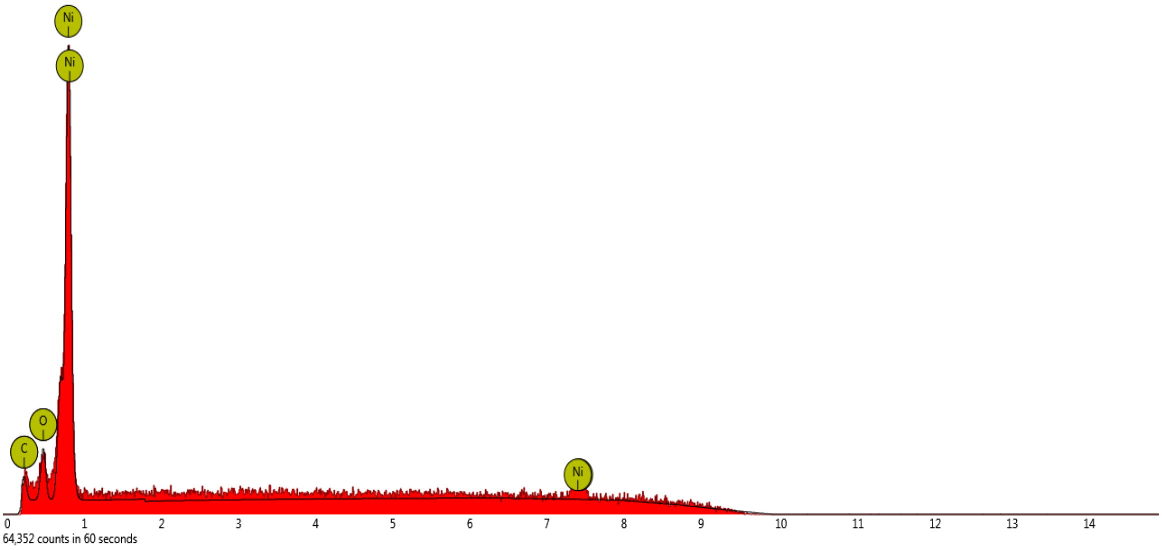

Element Number	Element Symbol	Element Name	Confidence	Concentration	Error
28	Ni	Nickel	50.0	83.6	1.1
8	O	Oxygen	Manual	6.3	4.0
6	C	Carbon	Manual	10.1	6.7

$Ni(OH)_2$(b)

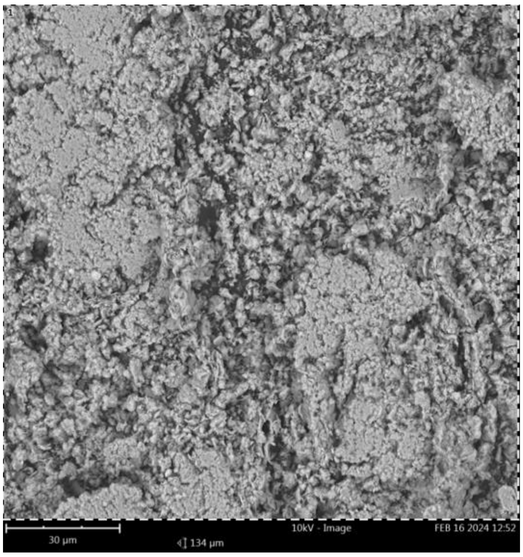

b)

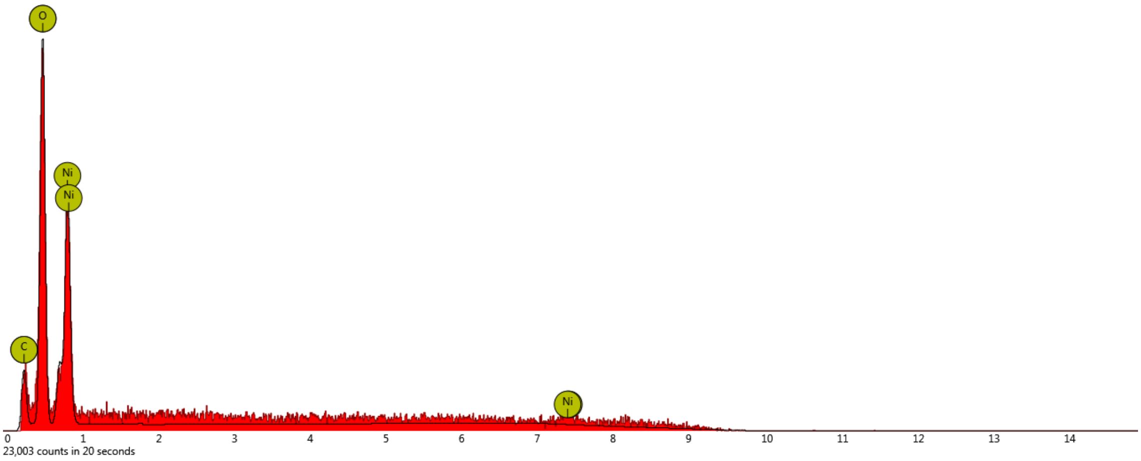

Element Number	Element Symbol	Element Name	Confidence	Concentration	Error
8	O	Oxygen	100.0	36.2	1.8
28	Ni	Nickel	50.0	49.5	2.2
6	C	Carbon	Manual	14.3	5.2

NiO400(c)

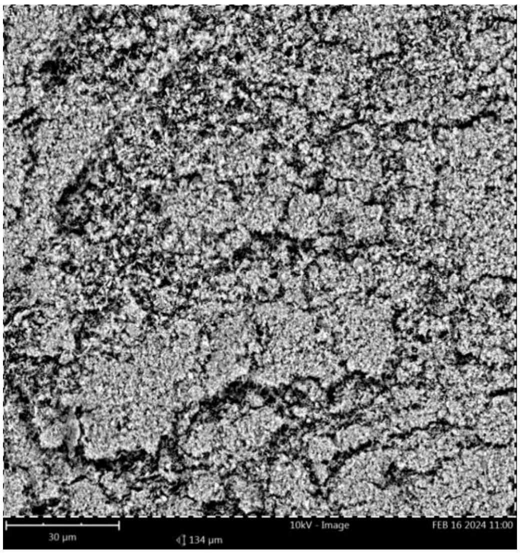

c)

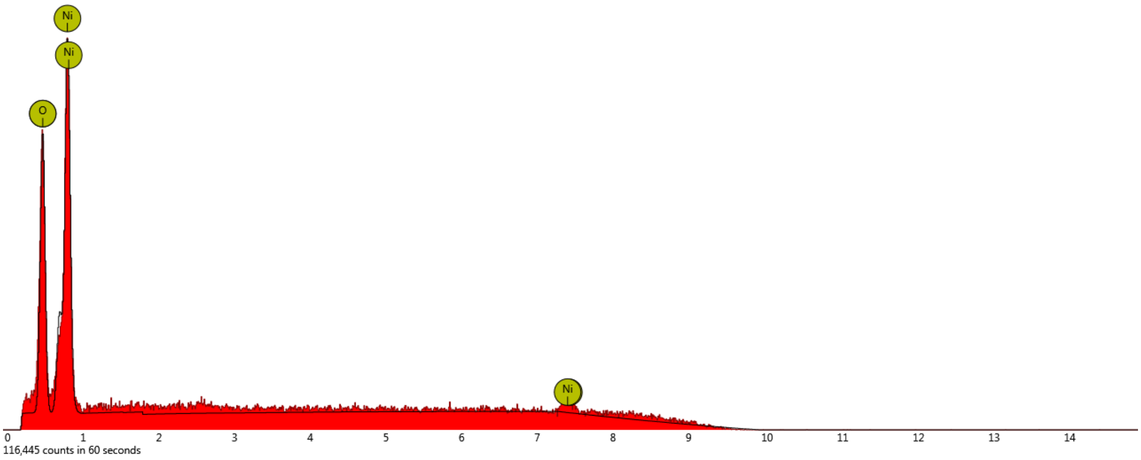

Element Number	Element Symbol	Element Name	Confidence	Concentration	Error
28	Ni	Nickel	50.0	74.9	0.9
8	O	Oxygen	100.0	25.1	1.1

Figure S4. *EDX analysis of precatalysts a) Ni(a), b) Ni(OH)$_2$(b) and c)* NiO400*(c)***.**

a)

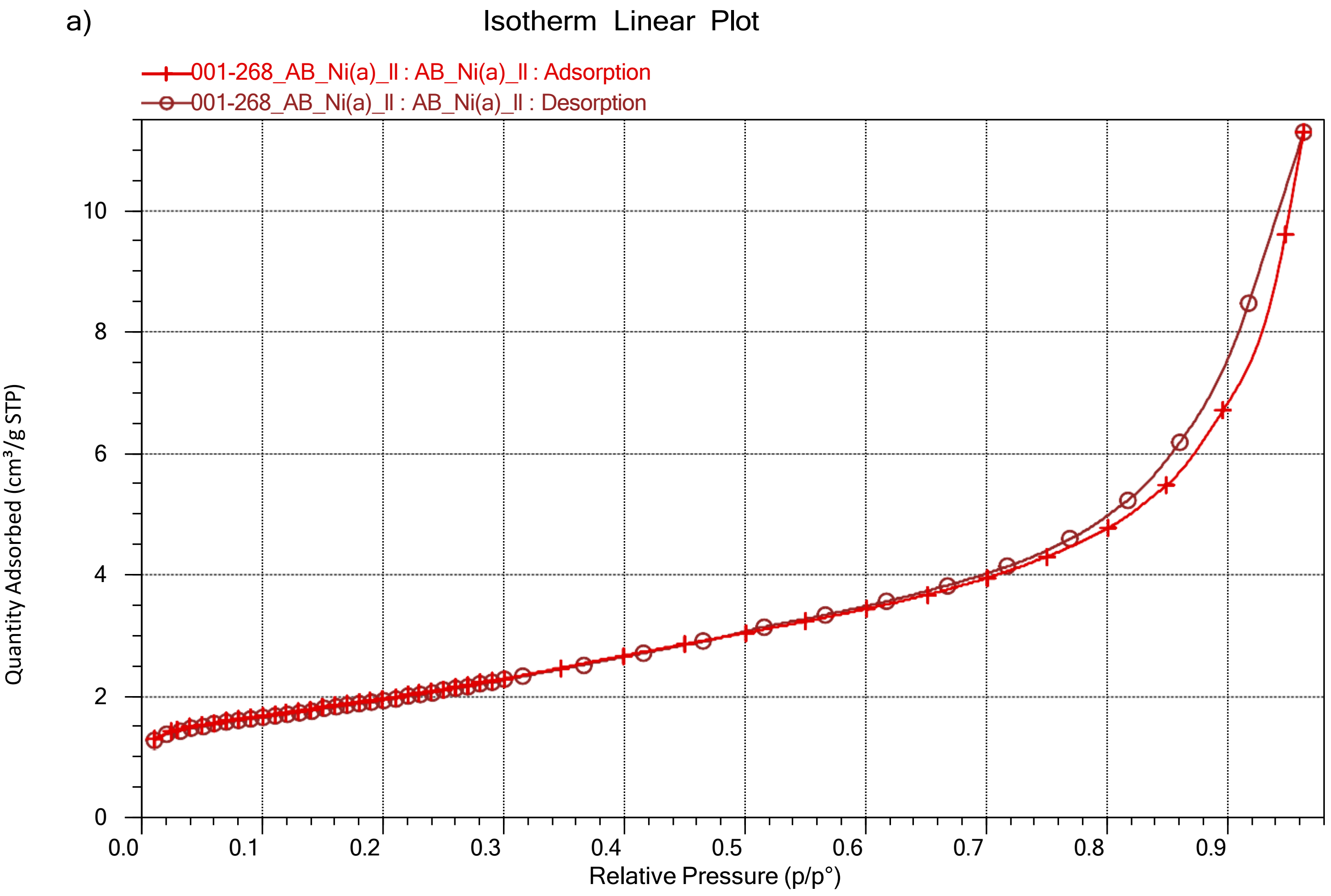

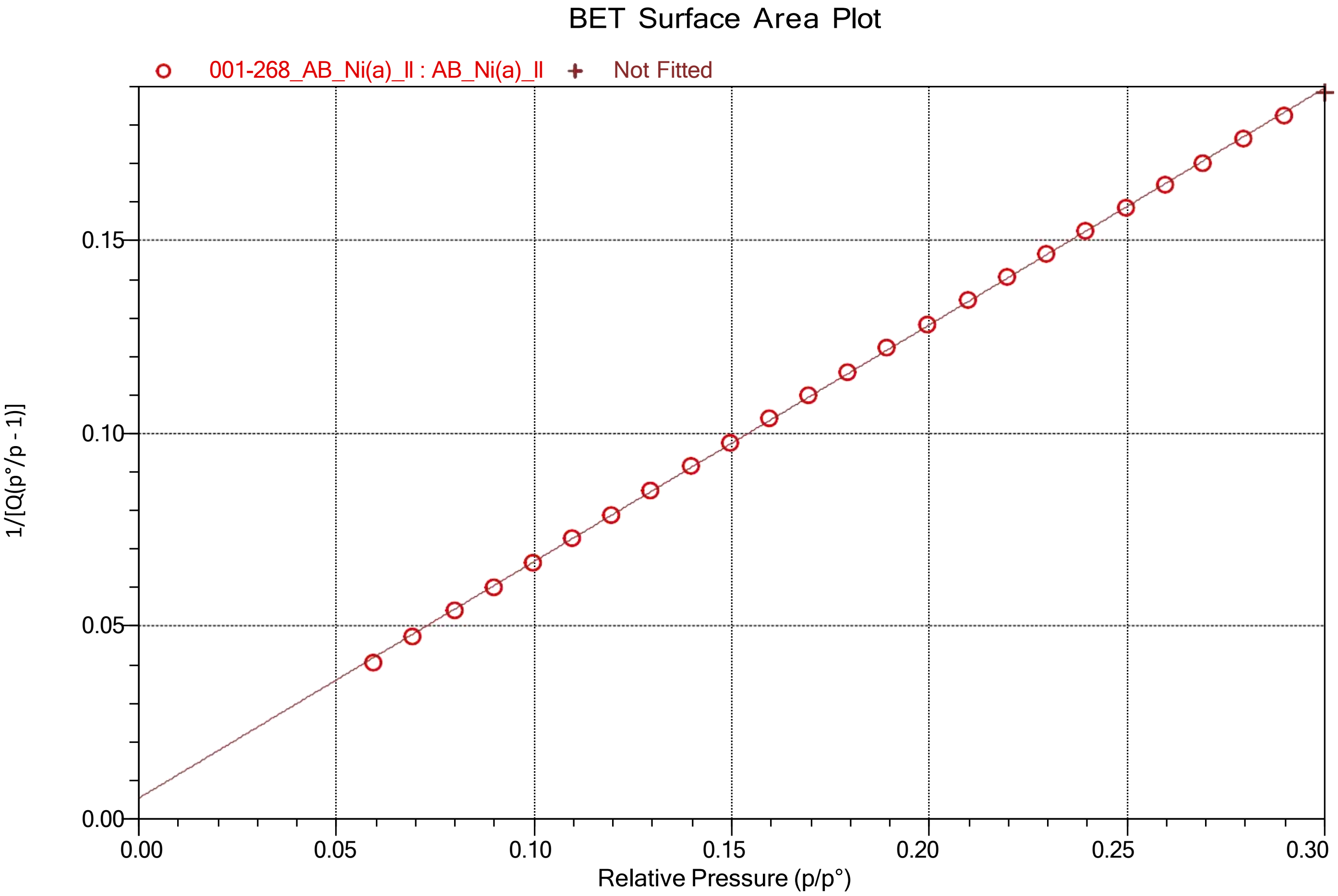

b) Isotherm Linear Plot

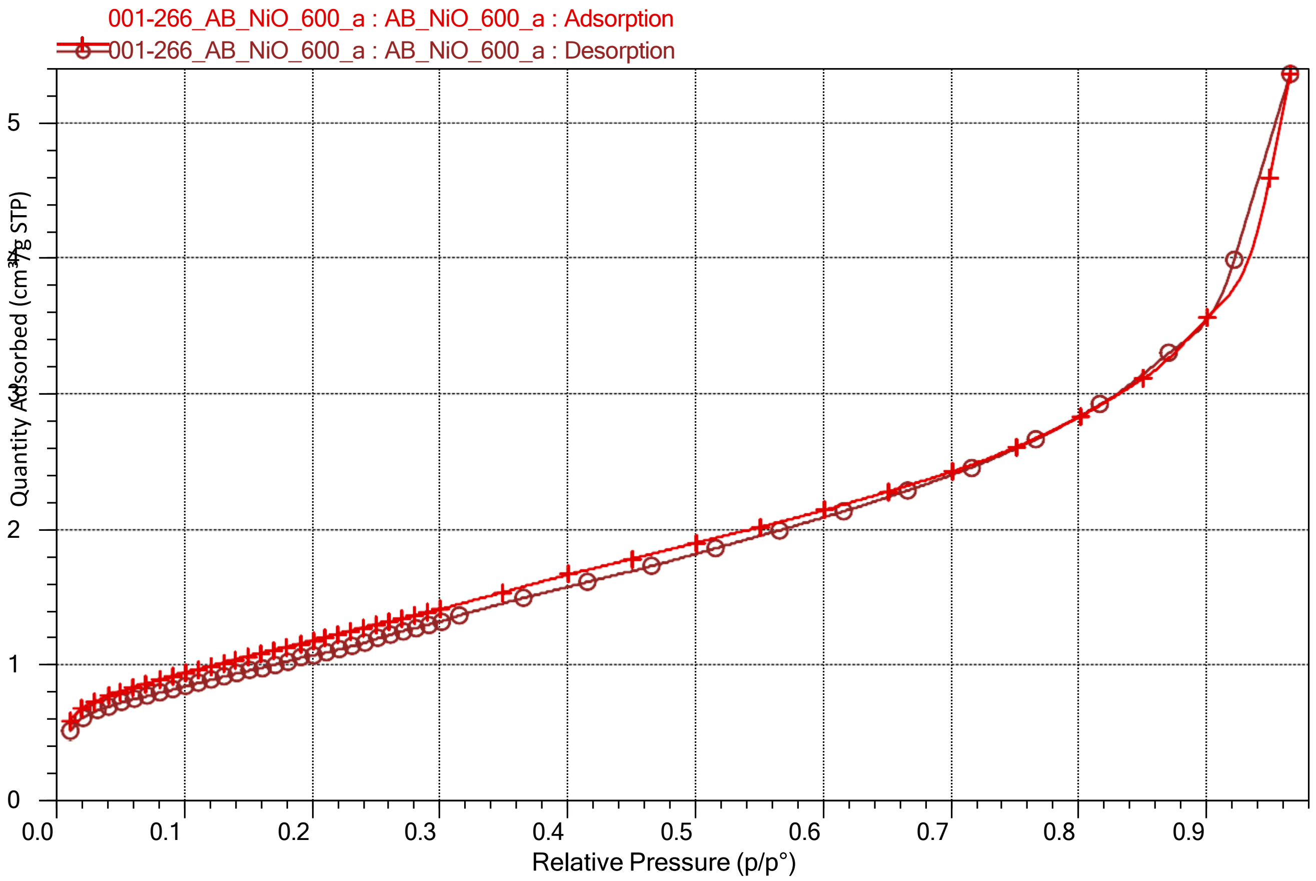

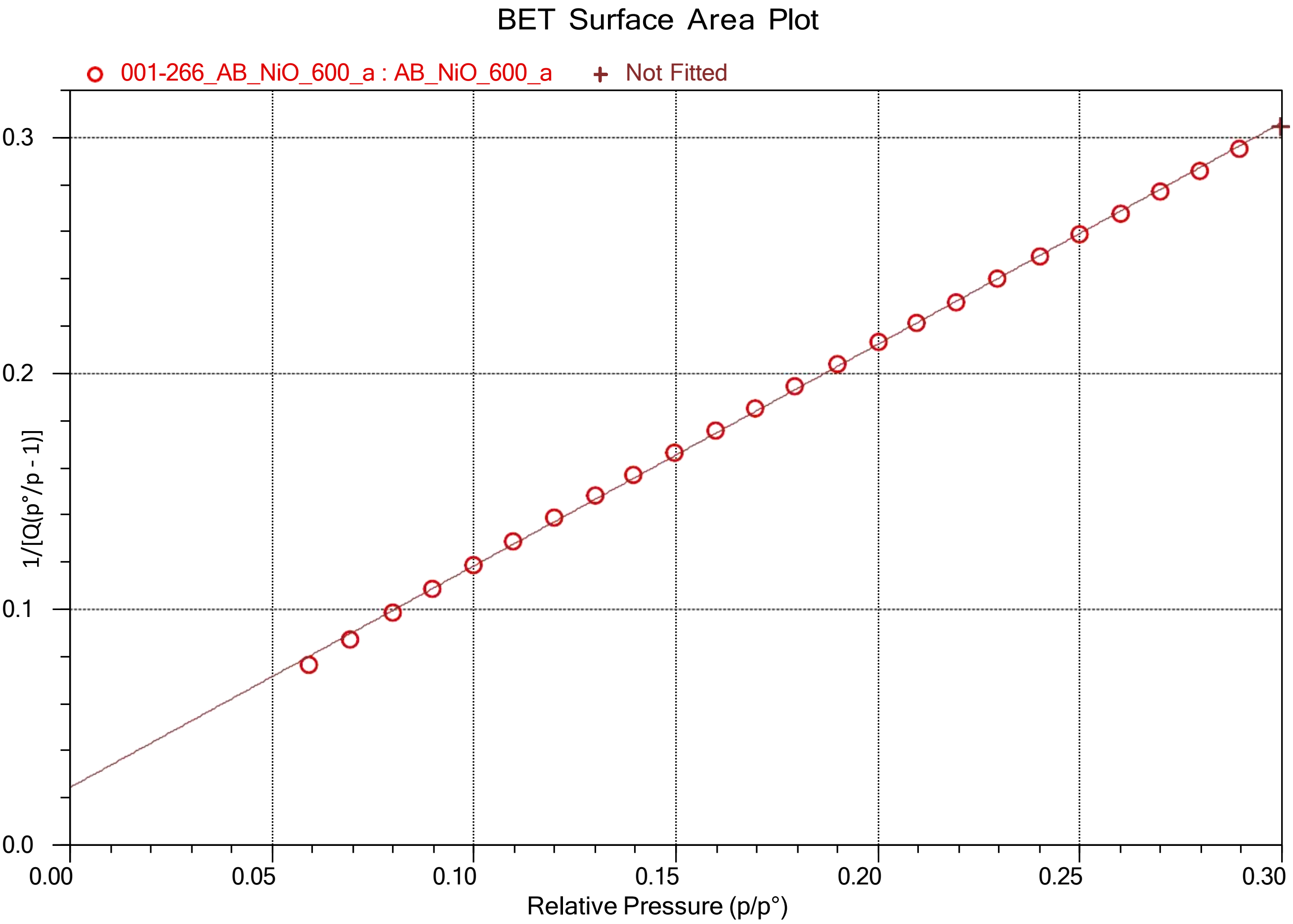

***Figure S5**. Isotherm Linear Plots and BET Surface Area Plots for a) Ni(a) and b) NiO600(a).*

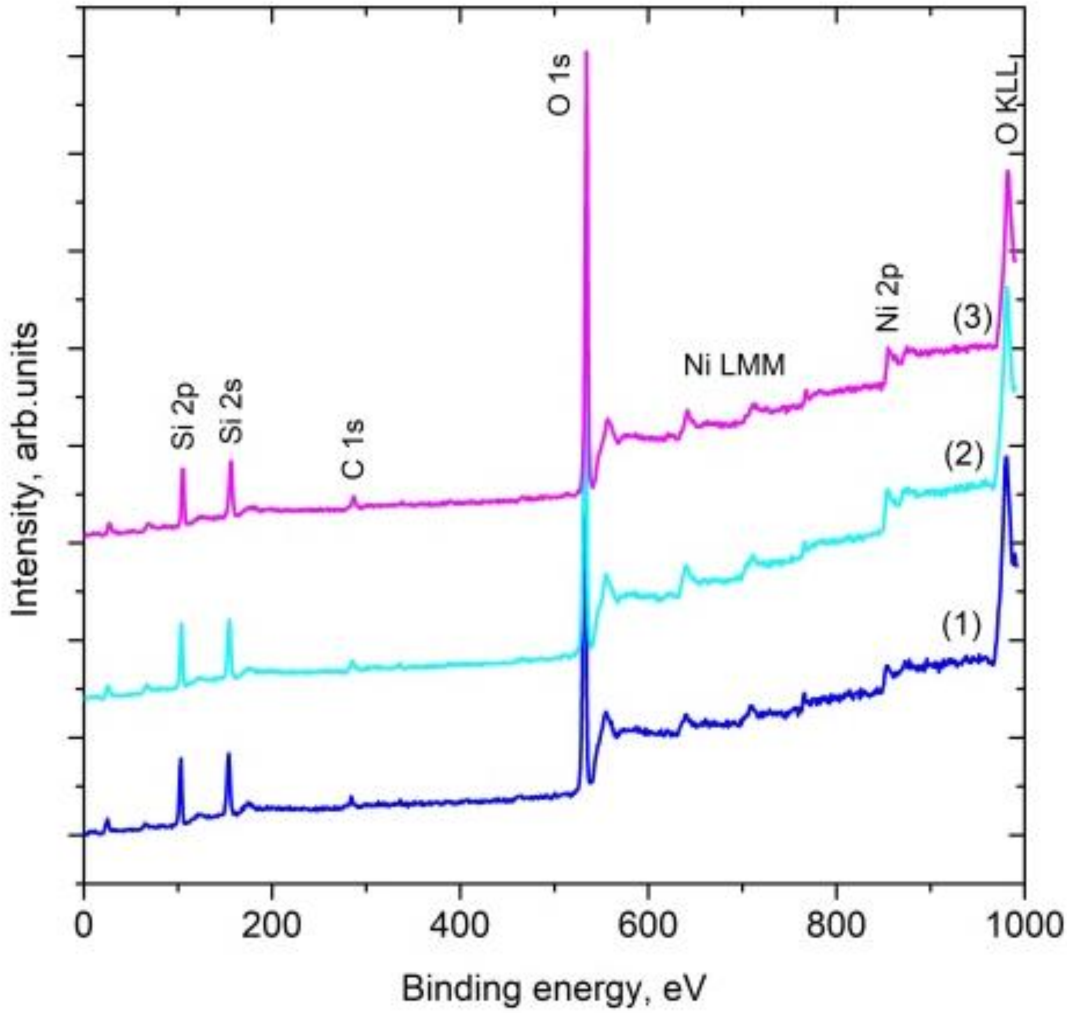

Figure S6 *X-ray photoelectron spectroscopy (XPS) survey spectra: Ni(a)* (1, blue), NiO400(b)* (2, light-blue), and Ni(OH)2(b)* (3, pink).*

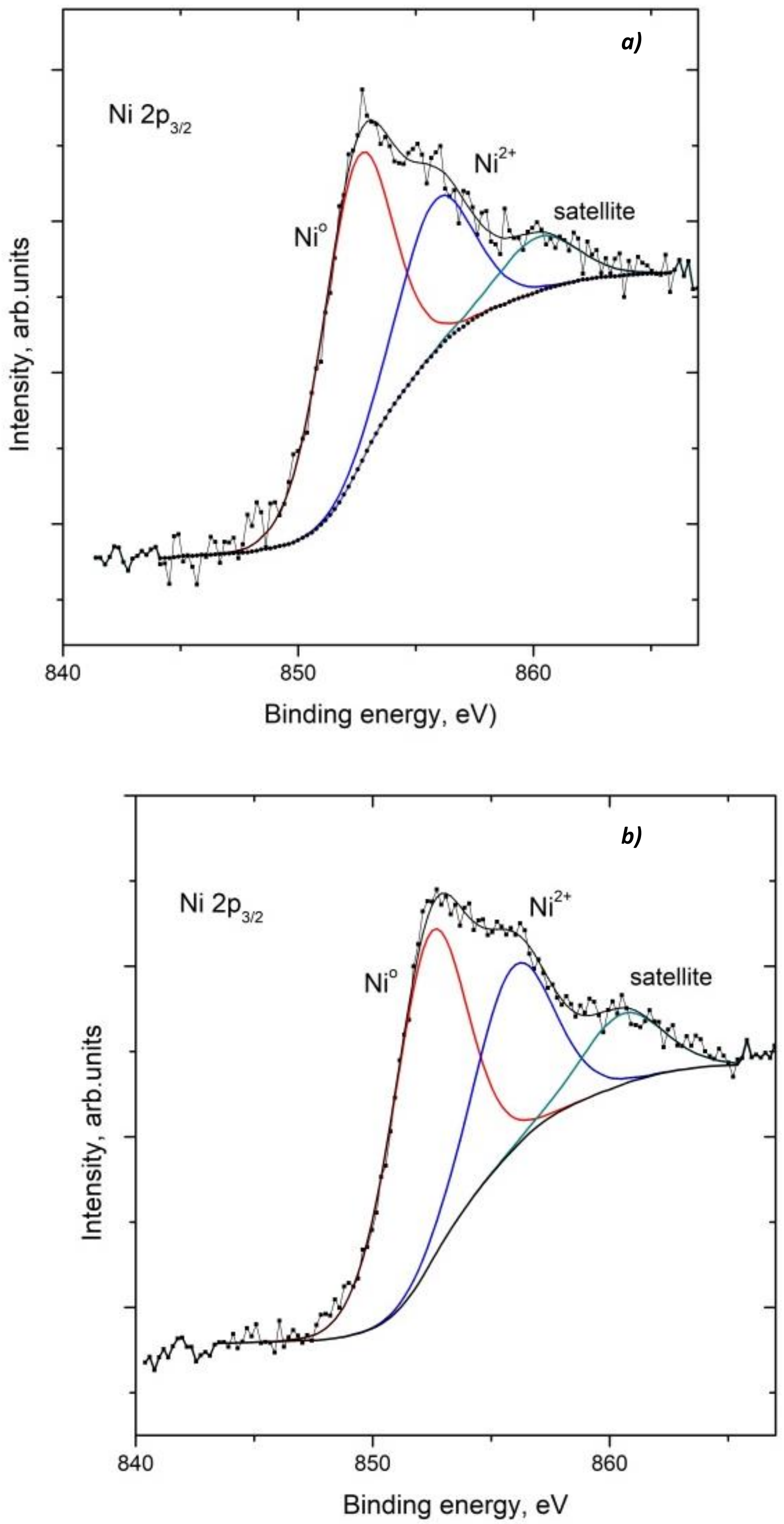

Figure S7 *Fitted spectra of Ni 2p*$_{3/2}$ *photoelectrons of (a) Ni(a)* and (b) NiO400(b)* catalyst samples.*

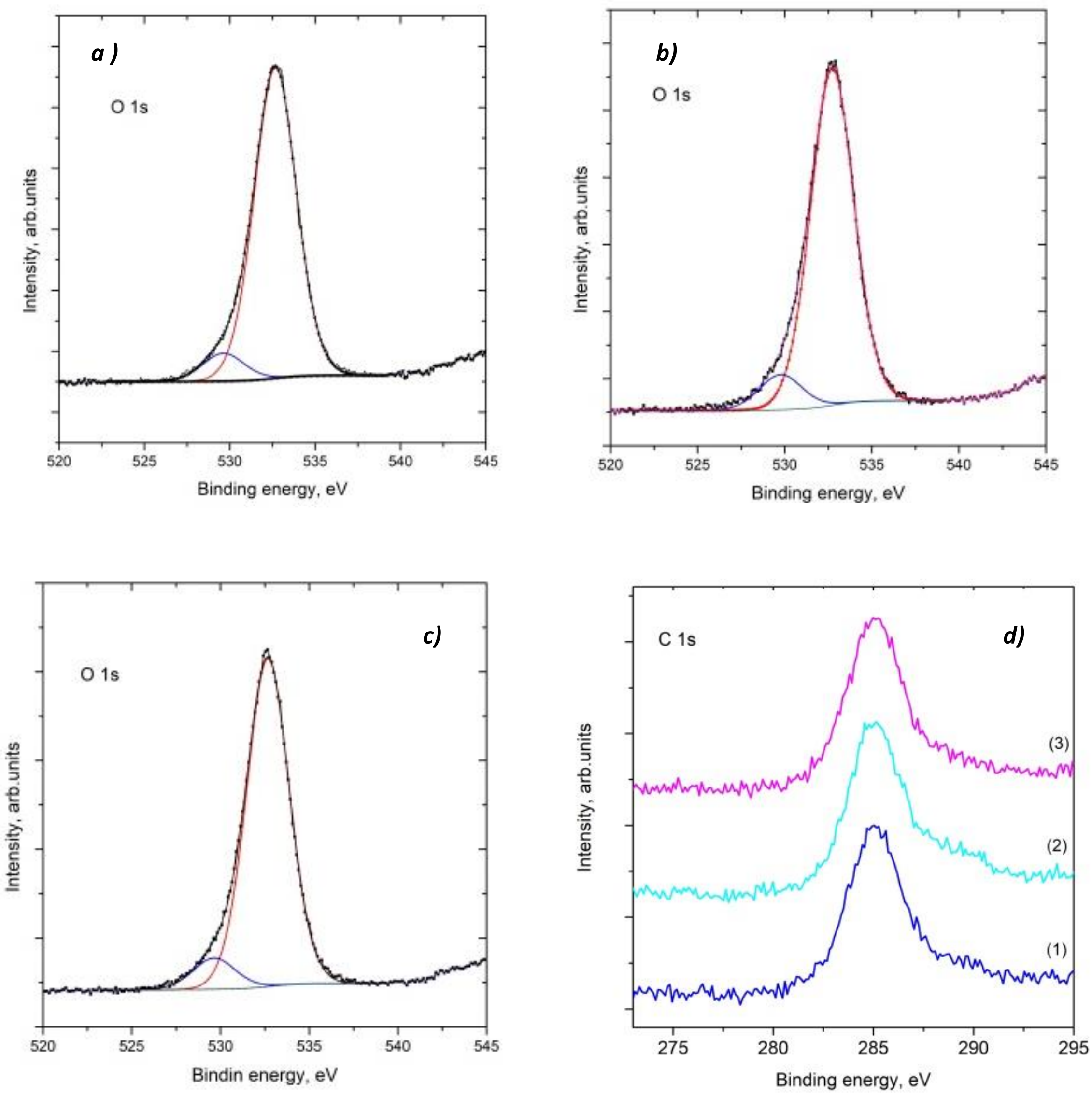

Figure S8 *Fitted spectra of O 1s photoelectrons of catalyst samples of (a) Ni(a)*, (b) NiO400(b)*, and (c) $Ni(OH)_2$(b)* catalysts and (d) of C 1s photoelectrons of Ni(a)* (1, blue), NiO400(b)* (2, light-blue), and Ni(OH)2(b)* (3, pink). Spectra are normalized to the same height and vertically shifted for the sake of clarity.*

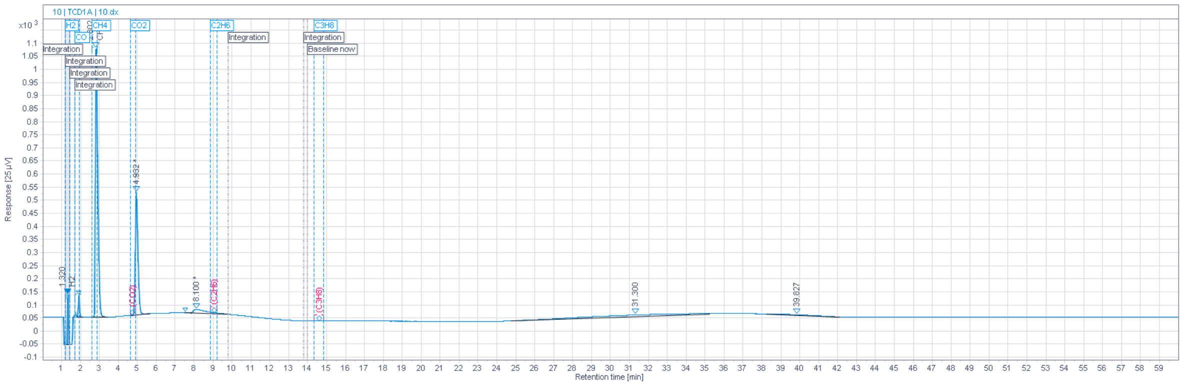
10 | TCD1A | 10.dx
H2
CO
CH4
CO2
C2H6
C3H8
Integration
Baseline now
1.320
4.932
8.100
31.300
39.627
Response [25 µV]
Retention time [min]

a)

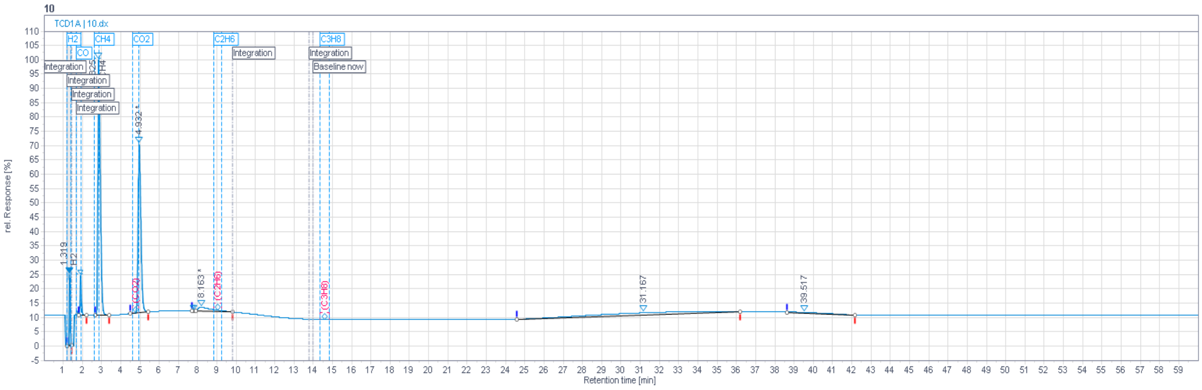
10
TCD1A | 10.dx
H2
CO
CH4
CO2
C2H6
C3H8
Integration
Baseline now
1.319
4.932
8.163
31.167
39.517
rel. Response [%]
Retention time [min]

b)

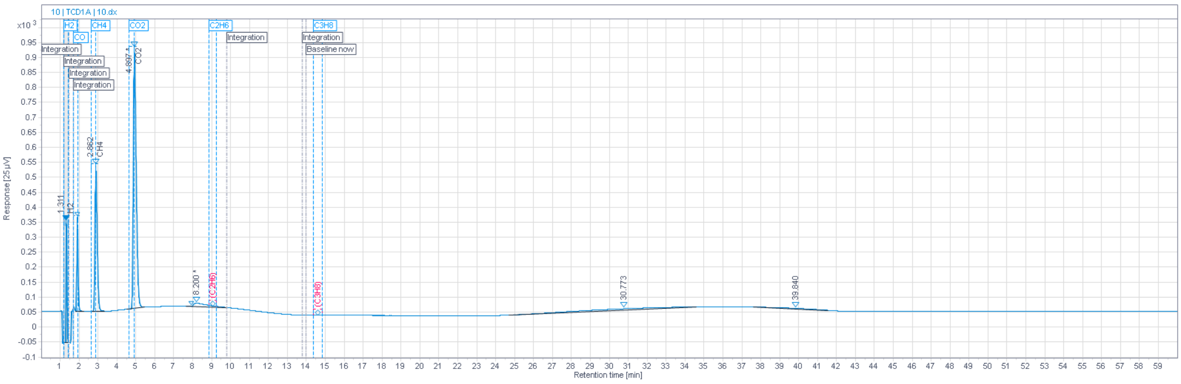
10 | TCD1A | 10.dx
H2
CO
CH4
CO2
C2H6
C3H8
Integration
Baseline now
1.311
2.862
4.897
8.200
30.773
39.840
Response [25 µV]
Retention time [min]

c)

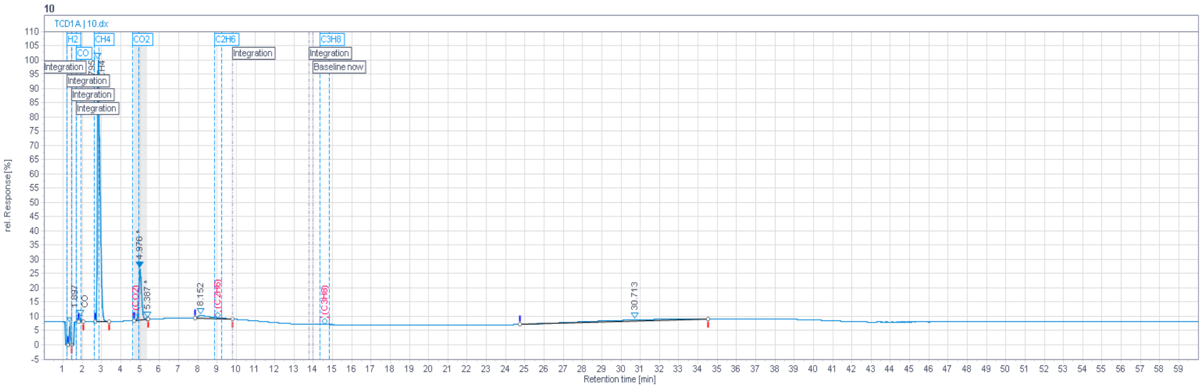
10
TCD1A | 10.dx
H2
CO
CH4
CO2
C2H6
C3H8
Integration
Baseline now
1.897
4.976
5.387
8.152
30.713
rel. Response [%]
Retention time [min]

d)

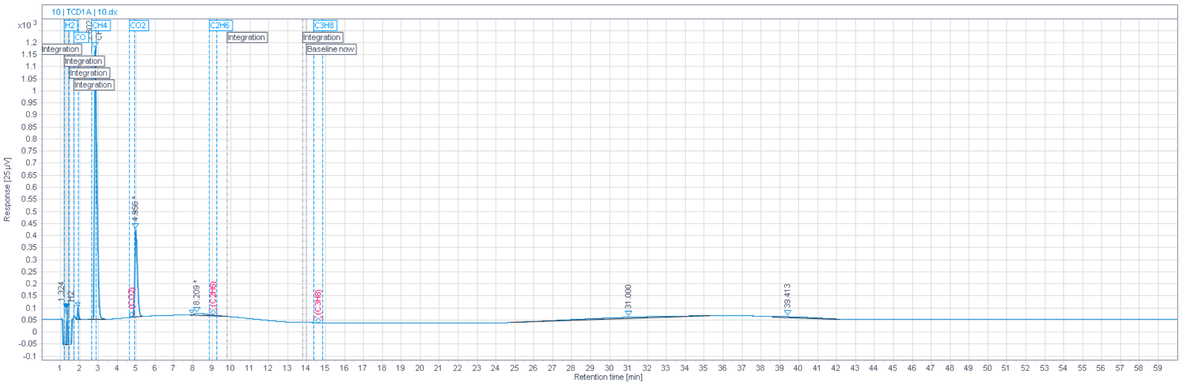
10 | TCD1A | 10.dx
H2
CO
CH4
CO2
C2H6
C3H8
Integration
Baseline now
Response [25 µV]
x10 3
4.956 *
8.209 *
31.000
39.413
Retention time [min]
e)

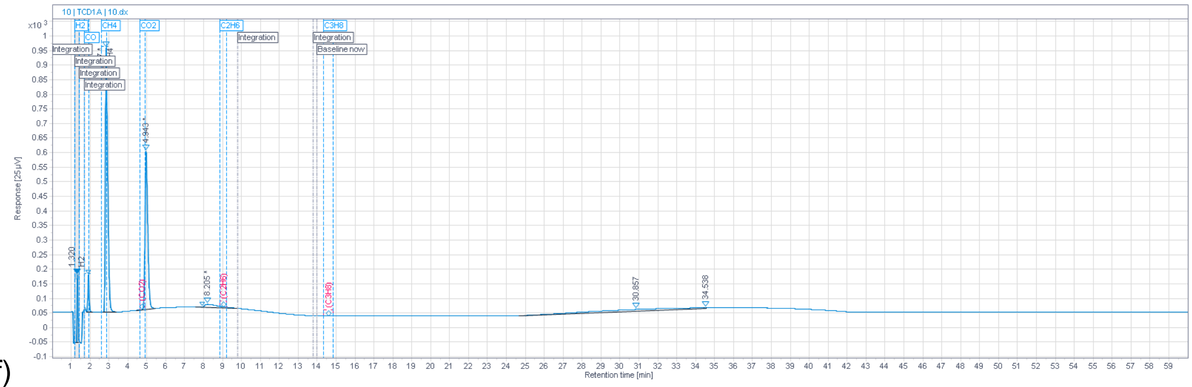
10 | TCD1A | 10.dx
H2
CO
CH4
CO2
C2H6
C3H8
Integration
Baseline now
Response [25 µV]
x10 3
1.320
4.943 *
8.205 *
30.857
34.538
Retention time [min]
f)

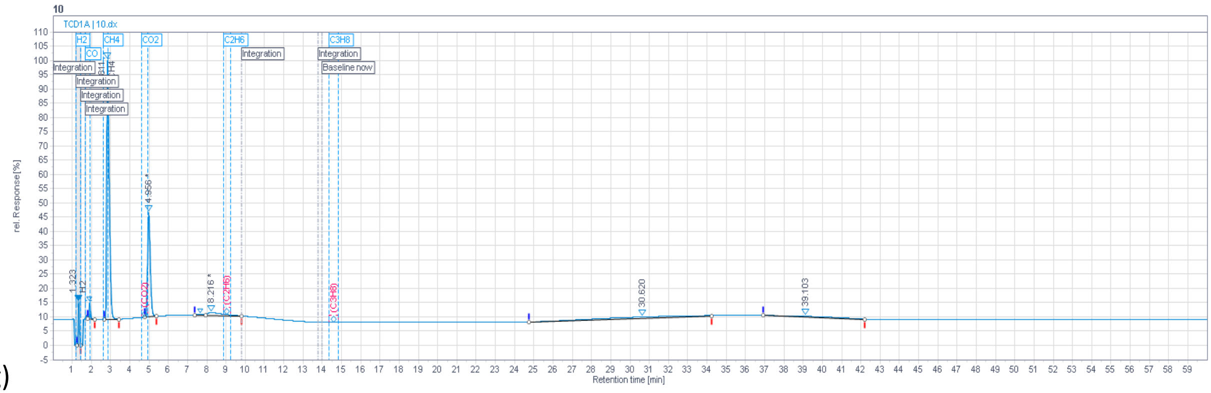
10
TCD1A | 10.dx
H2
CO
CH4
CO2
C2H6
C3H8
Integration
Baseline now
rel. Response [%]
1.323
4.956 *
8.216 *
30.620
39.103
Retention time [min]
g)

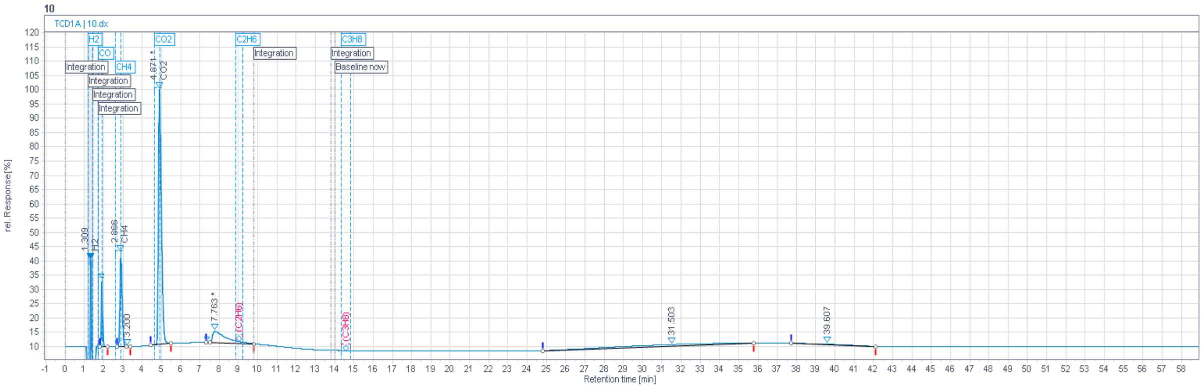
10
TCD1A | 10.dx
H2
CO
CH4
CO2
C2H6
C3H8
Integration
Baseline now
rel. Response [%]
1.309
2.886
3.200
4.871 *
7.763 *
31.503
39.607
Retention time [min]
h)

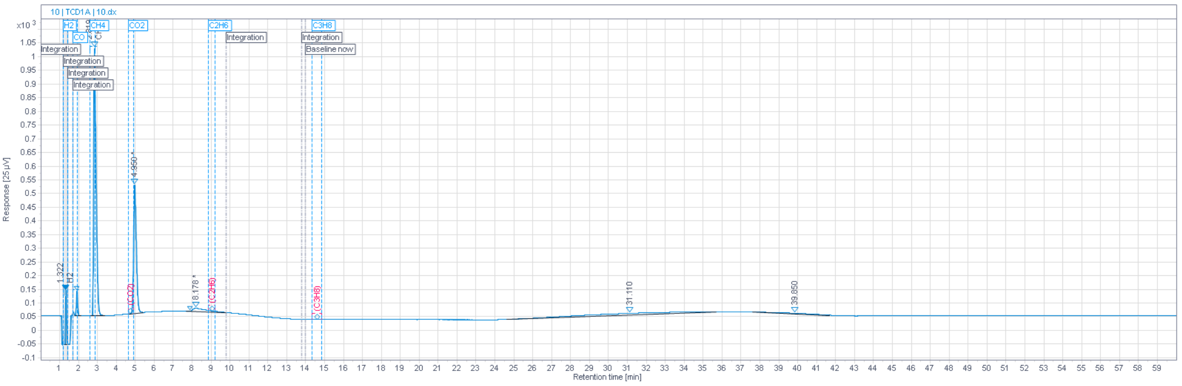

i)

Figure S9 *GC chromatograms taken during the catalytic experiments with Ni-consisted catalysts (10th hour): a) Ni(a), b) NiO600(a), c) NiO1000(a), d) Ni(OH)$_2$(b), e) NiO400(b), f) NiO600(b), g) NiO(OH)x(c), h) NiO400(c) and i) NiO600(c)*

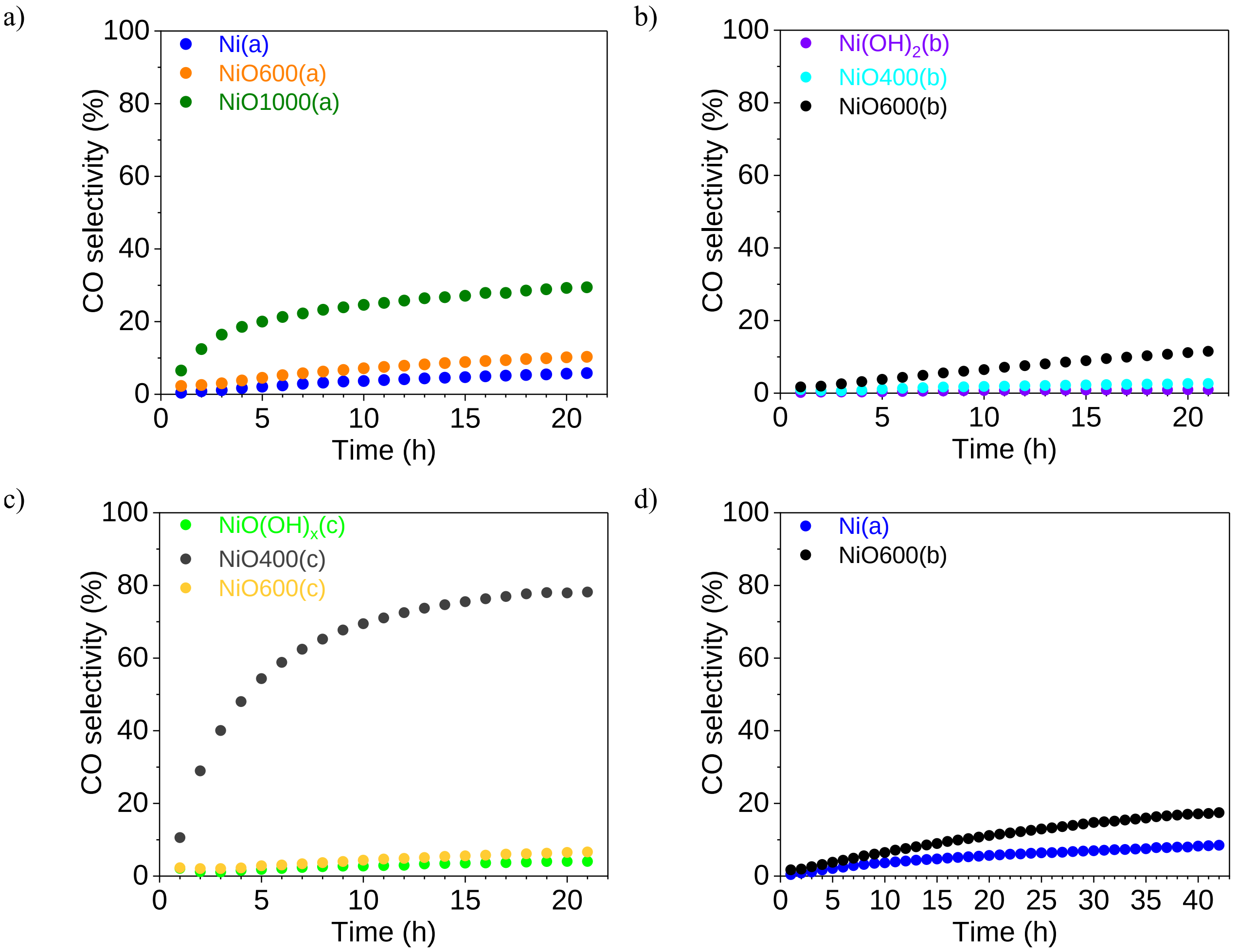

Figure S10 *Selectivity CO for a-group (a), b-group (b), c-group (c) of catalysts and in stability test (d).*

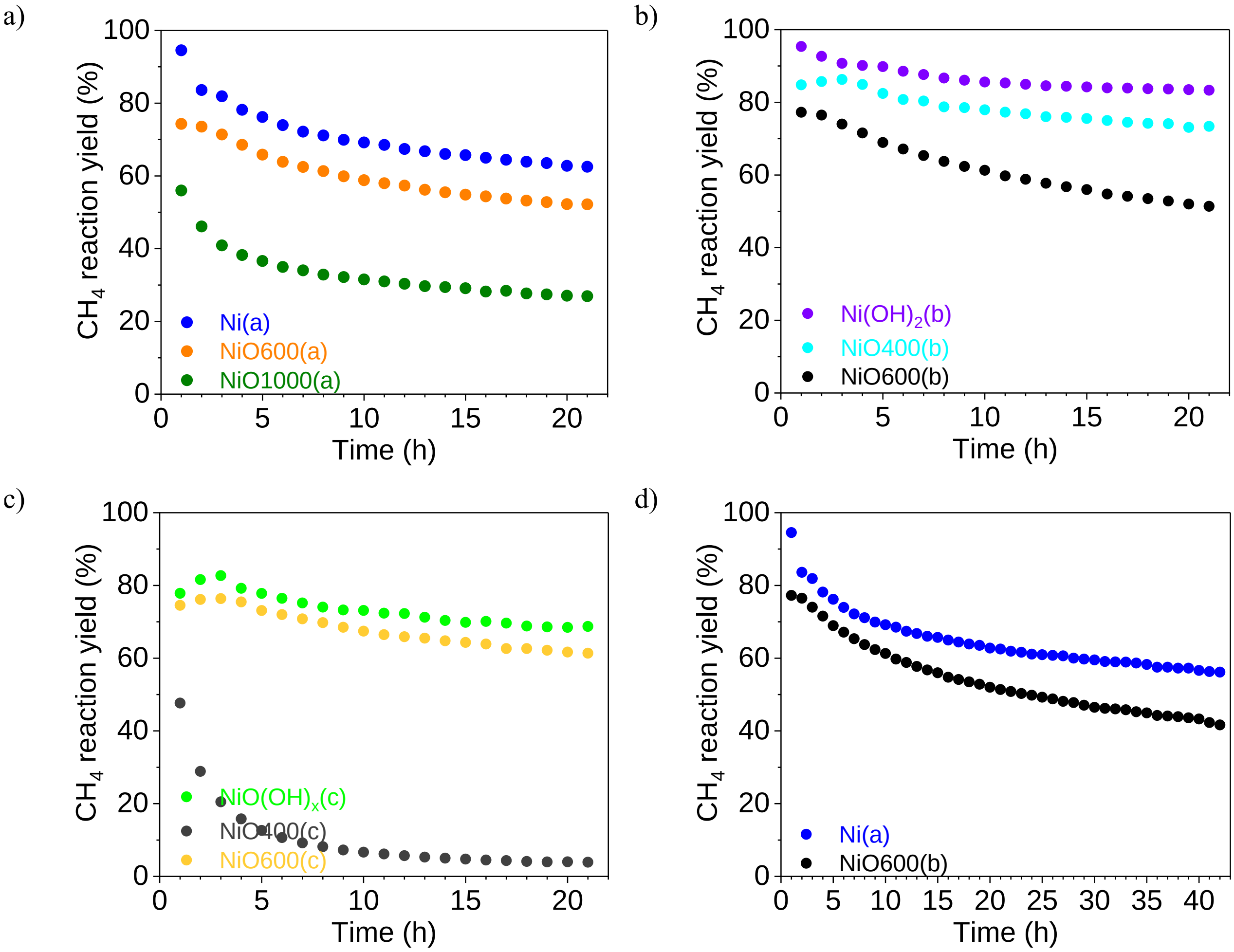

Figure S11 *Reaction yield for a-group (a), b-group (b), c-group (c) of catalysts and in stability test (d).*

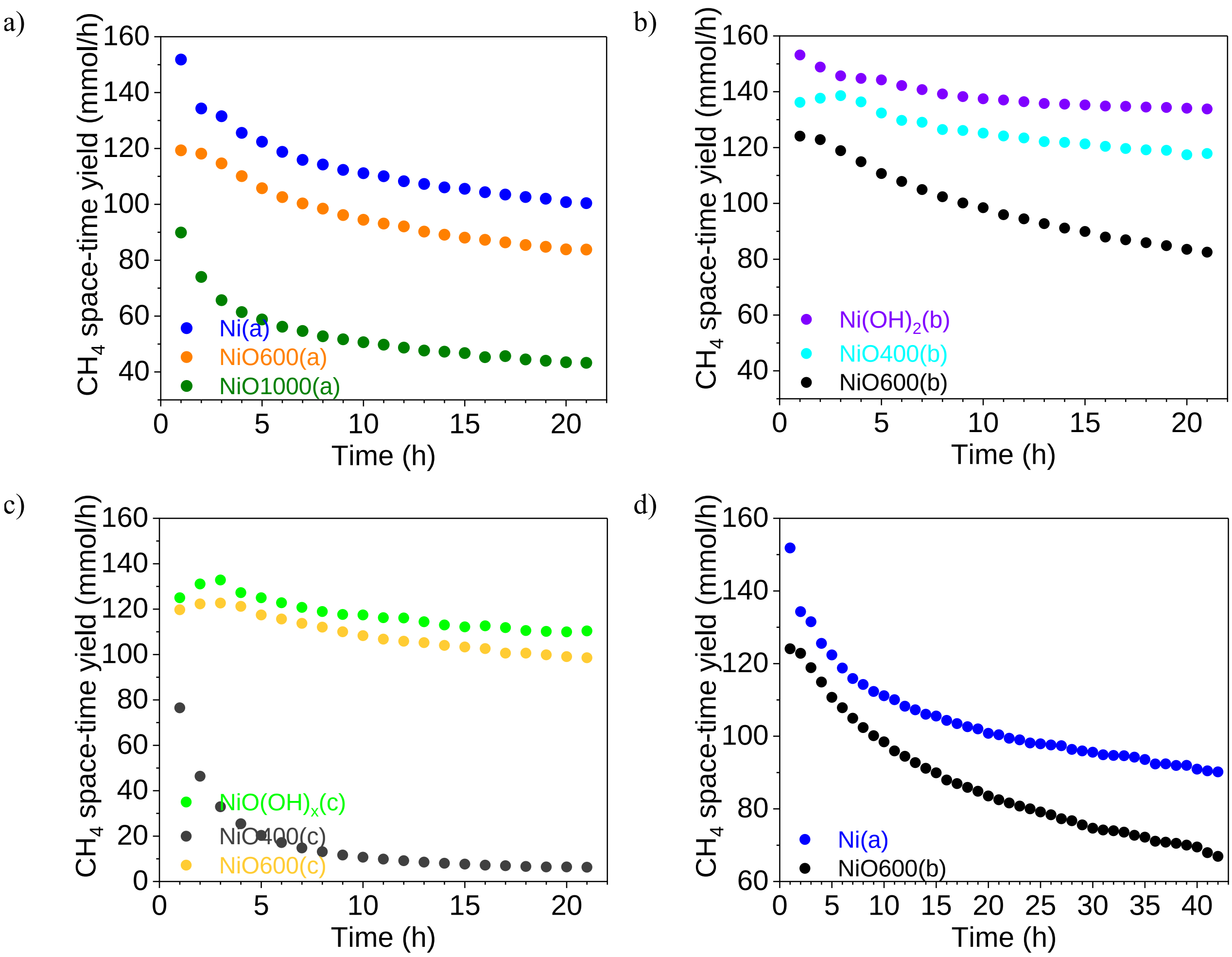

Figure S12 *Space-time yield for a-group (a), b-group (b), c-group (c) of catalysts and in stability test (d).*

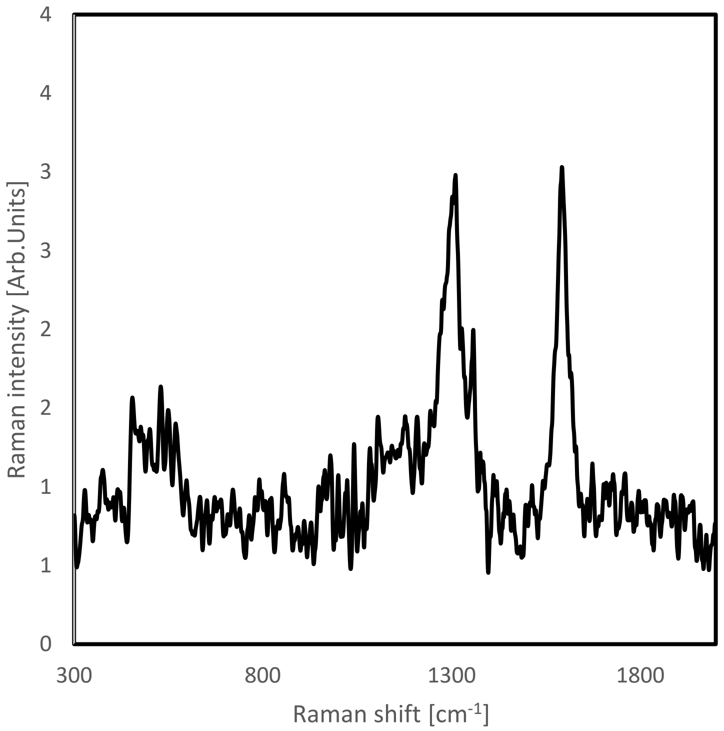

Figure S13 *Raman spectrum of $Ni(OH)_2$(b) sample, 780 nm, 10 mW.*
